# Single-cell phenotypic profiling and backtracing exposes and predicts clinically relevant subpopulations in isogenic *Staphylococcus aureus* communities

**DOI:** 10.1038/s42003-024-06894-z

**Published:** 2024-10-01

**Authors:** Jonathan Hira, Bhupender Singh, Tirthankar Halder, Anel Mahmutovic, Clement Ajayi, Arif Ahmed Sekh, Kristin Hegstad, Mona Johannessen, Christian S. Lentz

**Affiliations:** 1 Centre for New Antibacterial Strategies (CANS) and Research Group for Host-Microbe Interactions, Department of Medical Biology, UiT – The Arctic University of Norway, Tromsø, Norway; 2 Early Biometrics & Statistical Innovation Data Science & AI AstraZeneca, Biopharmaceuticals RD AstraZeneca, Mölndal, Sweden; 3 XIM University, Bhubaneshwar, India; 4 Norwegian National Advisory Unit on Detection of Antimicrobial Resistance, Department of Microbiology and Infection Control, University Hospital of North Norway, Tromsø, Norway

**Keywords:** Bacterial techniques and applications, Bacteriology

## Abstract

Isogenic bacterial cell populations are phenotypically heterogenous and may include subpopulations of antibiotic tolerant or heteroresistant cells. The reversibility of these phenotypes and lack of biomarkers to differentiate functionally different, but morphologically identical cells is a challenge for research and clinical detection. To overcome this, we present ´Cellular Phenotypic Profiling and backTracing (CPPT)´, a fluorescence-activated cell sorting platform that uses fluorescent probes to visualize and quantify cellular traits and connects this phenotypic profile with a cell´s experimentally determined fate in single cell-derived growth and antibiotic susceptibility analysis. By applying CPPT on *Staphylococcus aureus* we phenotypically characterized dormant cells, exposed bimodal growth patterns in colony-derived cells and revealed different culturability of single cells on solid compared to liquid media. We demonstrate that a fluorescent vancomycin conjugate marks cellular subpopulations of vancomycin-intermediate *S. aureus* with increased likelihood to survive antibiotic exposure, showcasing the value of CPPT for discovery of clinically relevant biomarkers.

## Abstract (web summary)

Phenotypic profiling using fluorescent probes combined with cell sorting and single-cell derived growth analysis detects differential cultivability and antimicrobial susceptibility in isogenic, but phenotypically heterogenous bacterial populations.

## Introduction

Bacterial pathogens cause infections as populations comprising myriads of single cells, but traditional experimental microbiology techniques and diagnostic clinical laboratory routines have commonly focused on the analysis of bulk populations, not taking cellular individuality into account. Even within isogenic bacterial populations, cellular phenotypic heterogeneity is generated through intrinsic (e.g., stochastic gene expression, cell age) and external factors (microenvironment, cell-to-cell interactions)^1^ affecting diverse traits related to bacterial physiology and stress response^2^, antimicrobial susceptibility^3,4^, or virulence^5,6^. Heterogeneity may benefit cell populations through cooperative behaviors (division-of-labor^1,7^, sharing of extracellular resources^5,8^), as well as through generation of specialized cells with fitness advantages under adverse conditions (bet-hedging^1,5^). Alternatively, heterogeneity may result from necessary trait adjustments according to ´cellular vigor´^6^. An increasing body of literature documents the clinical relevance and complications for treatment elicited by subpopulation phenotypes, particularly during chronic infections^9–14^. This includes heteroresistant cells, i.e., cellular subpopulations in clonal isolates characterized by largely different sensitivity towards certain antibiotics^3,15,16^. It also includes non-replicating cells known as persister cells that survive antibiotic treatment without developing inheritable, genetic resistance^4,11,14,17,18^. After antibiotic treatment, persisters can revert to a proliferating and antibiotic-susceptible phenotype, cause relapse and chronicity of infection^19–21^, and promote the evolution of antimicrobial resistance^14,22^. Persister cells can occur spontaneously at low frequencies, persistence can also be triggered by stress conditions, such as starvation and other factors^4^. Another dormancy phenotype referred to as ´viable-but non-culturable (VBNC)´ cells was identified by observing a discrepancy between colony forming units (CFU) and cells classified as *alive* with the help of viability stains^23,24^. The degree to which these different dormancy states are identical, related, distinct, or even artificial, is a matter of ongoing controversy ^25–28^.

The presence of both dormant and heteroresistant variants is inferred retrospectively by their ability to grow under conditions where the bulk of the cells do not^4,17^. Since there are no biomarkers to differentiate and separate these growth phenotypes from functionally different bulk cells, our ability to study them is limited. Phenotypic heterogeneity has been visualized by introduction of fluorescent labels and fluorescent reporter strains, providing increased understanding of heterogeneity particularly at the level of gene regulation^6,29–31^. In addition, we and others have shown that fluorescent chemical probes are excellent tools to study phenotypic parameters at the single cell level and expose phenotypic heterogeneity within native, isogenic cellular populations^32–34^. Coupled to time-lapse quantitative microscopy in microfluidic systems such as the mother machine, fluorescent labels, and reporters are powerful tools to decipher transcriptional status and even correlate it with growth parameters^35–39^ allowing microscopic identification and tracking of single dormant cells^38^. One disadvantage of microfluidics systems is a relatively low throughput and the difficulty to purify/recover cells for follow-up studies beyond microscopy.

**Fig. 1:**
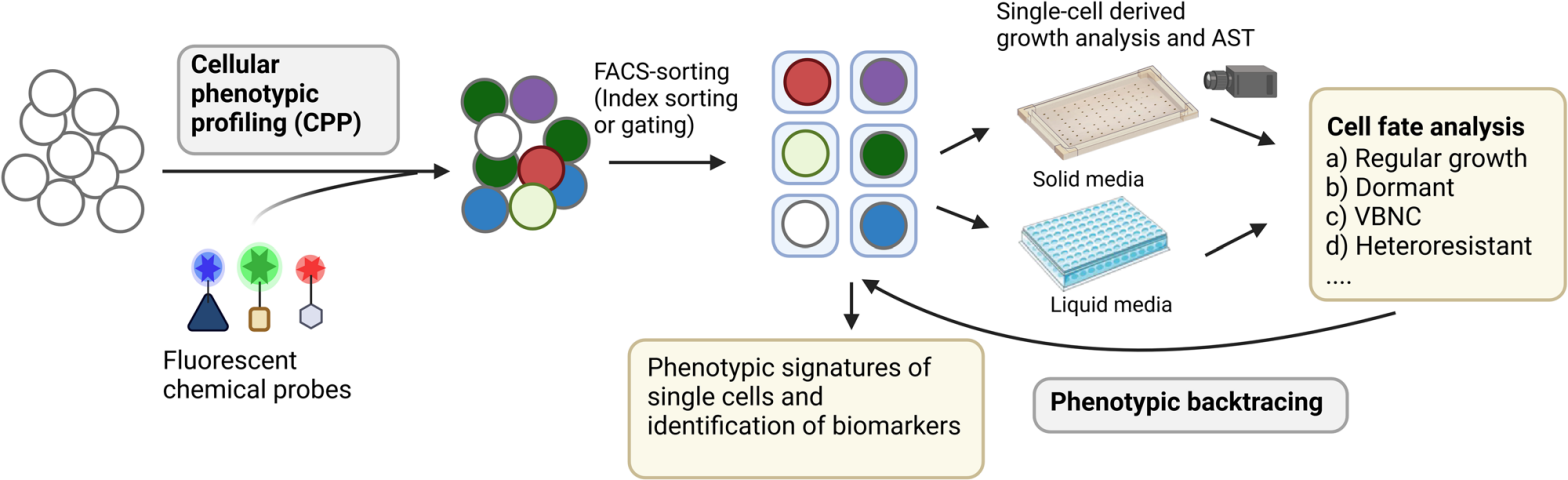
Overview of the ´Cellular phenotypic profiling and backtracing´ (CPPT) platform. Phenotypic traits in naïve bacterial cell populations will be visualized using fluorescent chemical probes and quantified by flow cytometry. Single cells are sorted out and fed into a downstream analysis of growth performance in liquid media, where replication is monitored in a plate reader, or onto agar, where growth is monitored by time-lapse imaging. After functional analysis, cell fates are classified into categories of interest and the phenotypic profile at the time of sorting is traced back, allowing for detection of phenotypic signatures associated with dynamic reversible phenotypes and discovery of biomarkers. The figure was created with BioRender.

To fully expose and understand cell individuality and cooperativity, a systematic framework is needed that combines visualization of diverse phenotypic traits with separation and broad functional characterization of phenotypically different cells. Inspired by pioneering studies utilizing flow cytometry-based enrichment of persisters^25,40–43^, we here present a fluorescence-activated cell sorting (FACS)-based platform for high-throughput (HT) single cell phenotypic profiling coupled to functional analysis of single bacteria (Fig. 1). In a first stage, bacteria with different phenotypic traits are differentiated through *CellularPhenotypicProfiling* (CPP) using fluorescent chemical probes, before single cells are sorted for separate analysis downstream and determination of their cell fate. In a feature we are referring to as *Phenotypic Backtracing*, the phenotypic profile of a cell at the time of sorting can be traced back after determining the cell fate post-sorting. In this proof-of-principle study focusing on the clinically relevant Gram-positive pathogen *S. aureus*, this combined platform, *Cellular Phenotypic Profiling and backTracing* (CPPT), readily detects growth variants such as non-stable small colonies and other bistable growth phenotypes by exposing the differential ability of single cells to outgrow in liquid or solid media. Finally, for a vancomycin-intermediate susceptible (VISA) strain known to produce a thickened cell wall decorated with decoy targets, using CPPT we demonstrate that a fluorescent vancomycin conjugate is a biomarker for cellular subpopulations with reduced susceptibility to vancomycin highlighting the potential of chemical probes for differentiating clinically relevant subpopulations.

## Results

### Coupling fluorescence-activated cell sorting of bacteria to single-cell derived growth analysis

**Fig. 2:**
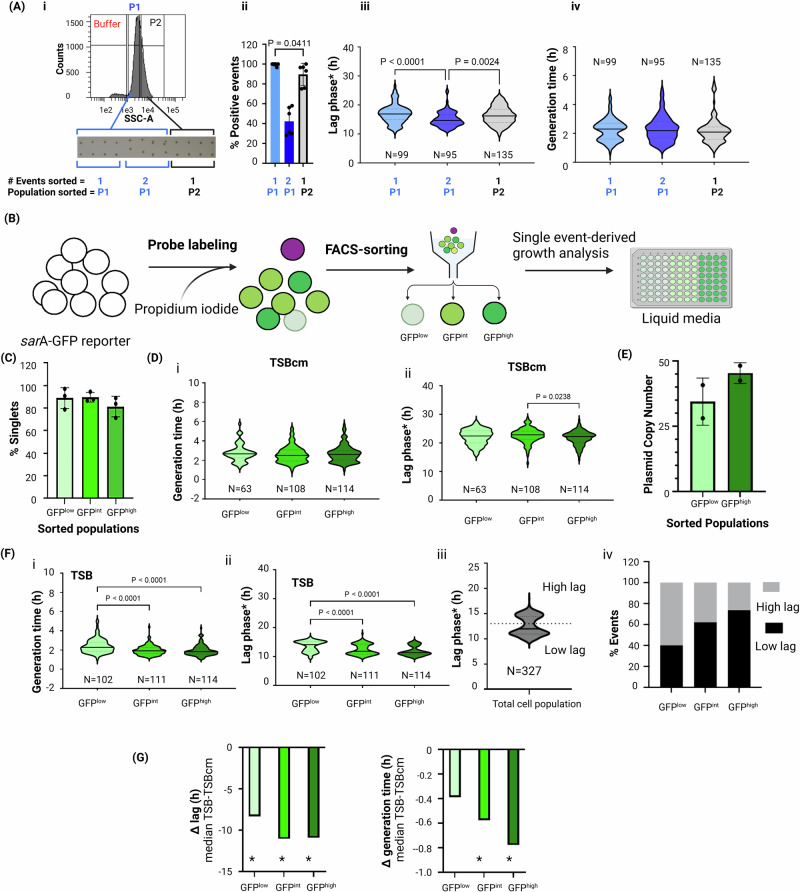
Evaluation of single-cell sorting in a *sar*A-GFP reporter strain of *S. aureus.* **A** Gating scheme for sorting single *S. aureus* cells followed by growth analysis, (i) SSC-A histogram of *S. aureus* cells showing buffer noise (red) and the two cell populations SSC^low-P1^ (blue) and SSC^high-P2^ (black). The inset below shows CFUs on solid media following indicated sorted events from gates SSC^low-P1^ and SSC^high-P2^, (ii) Percentage of positive events (single or double CFUs) on TSAcm solid media (average ± standard deviation (SD)) of 2 independent colonies (pre-sorting cultures). Statistical comparison was performed between single sorted events from subpopulations SSC^low-P1^ and SSC^high-P2^, (iii) Violin plot showing lag phase* (h), and (iv) generation time (h) of indicated sorted events in TSBcm liquid media with *N* representing 95 to 135 post-sorting cultures derived from 2 independent pre-sorting cultures. Detailed description of gating scheme is presented in Supplementary Fig. 2C, D, **B** Schematic overview of the experimental set up of sorting single events from SSC^low-P1^ subpopulation based on their GFP profile. Cells were stained with PI and sorted into GFP^low^, GFP^int^ and GFP^high^ PI^-^ subpopulations. Detailed description of gating scheme is presented in Supplementary Fig. 2C, D, **C** Imaging flow cytometry analysis of singlet percentages in GFP^low^, GFP^int^ and GFP^high^ subpopulations. The graph shows average ± SD from 3 independent biological replicates. The data were not significantly different. **D** Growth analysis of single cells sorted from GFP^low^, GFP^int^ and GFP^high^ subpopulations in TSB media containing 10 µg/ml chloramphenicol (TSBcm) (i) Generation time and (ii). Lag phase* (h) duration. The graph shows violin plots generated from *N* representing 63 to 114 individual cultures derived from 2 independent colonies (pre-sorting cultures). **E** qPCR quantification of the average *sar*A-GFP reporter plasmid pCM29 copy number per cell derived from GFP^low^ and GFP^high^ subpopulations (*n* = 2 replicates). Data were not significantly different. **F** Growth analysis of single cells sorted from GFP^low^, GFP^int^ and GFP^high^ subpopulations in antibiotic-free TSB media (i) Generation time (ii). Lag phase* (h) duration of individual single-cell derived cultures (*N* = 102 to 114) from 2 independent colonies (pre-sorting cultures). (iii) Violin plot showing the lag phase distribution of the total sorted cell population (i.e., combined data from GFP^low^, GFP^int^ and GFP^high^ subpopulation). The dotted line (at 13 h) shows the threshold cell for separation of cell fates into´high lag´ and ´low lag categories. (iv) Percentage of events in the ´high lag´ and ´low lag´ cell fate categories for the GFP^low^, GFP^int^ and GFP^high^ subpopulations. **G** Burden associated with plasmid carriage quantified in terms of median decrease in (i) lag phase* duration and (ii) generation time in the absence of antibiotic selection pressure. These data were obtained by subtracting lag phase*/generation time values obtained from TSBcm values from those determined upon growth on TSB, as shown in Fig. 2D, F, respectively. Significant differences between TSBcm and TSB are indicated with *. Calculations and corresponding P values are presented in Supplementary Data 1. Statistical analysis in **A**, **C**, **D**, **F**, and **G** were performed by Kruskal–Wallis test in combination with Dunn´s multiple comparison test, and in **E** with two-tailed Mann–Whitney test. *N* represents sample size in violin plots. Only significance differences are shown and represented as *P*-values. Cross lines in all violin plots represent median values. Raw data and calculations are available in Supplementary Data 1. The figure was created with BioRender.

We chose to use bacterial colonies, as they represent ideal model communities comprising bacteria differing in age, availability of nutrients, and exposure to other microenvironmental parameters that induce phenotypic heterogeneity in e.g., gene expression, replication status, or antibiotic tolerance^44–47^. Since *S. aureus* is well known for its ability to aggregate and to form grape-like structures, we first aimed to determine whether it was possible to reliably sort single *S. aureus* cells by FACS. For method establishment, we aimed to employ a fluorescent reporter strain that gives a robust and constitutive fluorescent signal that would facilitate distinction of bacteria from buffer noise/smaller contaminating particles. We therefore employed a plasmid-based transcriptional reporter strain producing Green Fluorescent Protein under control of the P1 promoter of the *sar*A gene^48^ which encodes a global regulator of *S. aureus* virulence genes^49^ (Plasmid map in Supplementary Fig. 1). Additionally, cells were labeled with propidium iodide (PI) to exclude dead cells. We found that the most commonly used flow cytometry parameter for cell size, forward scatter (FSC), failed to discriminate individual *S. aureus* cells from background signals and showed a wide FSC distribution (Supplementary Fig. 2B-iii, 2C-iii). However, the log side-scatter (SSC) profile reliably resolved submicron-sized beads (Supplementary Fig. 2A), and *S. aureus* cells showed a narrow distribution distinct from buffer noise (Supplementary Fig. 2B-iv, 2C-iv). To test if those cellular events with a higher SSC-signal might include aggregates as indicated by SSC-H vs SSC-A correlation plot (Supplementary Fig. 2B-v, 2C-v), we sorted SSC^low^ (subpopulation P1) and SSC^high^ (subpopulation P2) events onto solid media for determination of CFU following overnight incubation. We observed that the SSC^low-P1^ population yielded >99% ± 1.4 (av. ± sd) single CFUs and the SSC^high-P2^ population contained >89% ± 11.16 single CFUs (Fig. 2A-i, A-ii). Whereas the cell number can be readily determined as CFUs on solid media, in liquid culture the number of inoculated bacteria affects the lag phase^50^. We therefore sorted single or multiple events into 96-well plates with liquid media and recorded growth curves, and recorded the time taken by cultures to surpass the minimal threshold of detection in the plate reader (referred to as lag phase*), and calculated the generation time, which for single-event derived cultures were surprisingly heterogeneous. Yet, we observed an expected inoculum-dependent decrease in lag phase* (Supplementary Fig. 3C, 3D)^51^. To rule out that the FACS-sorting harms the cells and affects the outgrowth performance (e.g., through exposure to laser), we compared the growth kinetics of single and consortia of sorted cells with cultures achieved by serial dilution of batch cultures. The growth kinetics of cultures with equal numbers of cells derived by the two methods were comparable, demonstrating that FACS-sorting does not impact the cell growth kinetics (Supplementary Fig. 3E). Lag phases* derived from either single or two sorted events (from the SSC^low-P1^ population) were overlapping due to the high level of heterogeneity, but statistically different (*P* < 0.0001; Fig. 2A-iii). The lag phase* from single SSC^high-P2^ population was not different to that of single SSC^low-P1^ population, but significantly higher than that achieved from cultures derived from two SSC^low-P1^ events (*P* = 0.0021), suggesting that any putative cell aggregates in this SSC^high-P2^ population either have growth characteristics indistinguishable from single CFUs or their abundance in the population is too low to affect the statistical analysis between the groups.

These data suggest that we can localize and sort single CFUs, but we cannot rule out that a CFU may be constituted by two or more cells. To address this, we separated live SSC^low-P1^ cells based on *sar*A-GFP reporter activity (GFP^+^, PI^–^) into three subpopulations, GFP^low^, GFP^int^ and GFP^high^ (Fig. 2B, Supplementary Fig. 2D) and sorted ~60,000 cells per gate for imaging flow cytometry. For all subpopulations >80% of sorted events were single cells, <20% were doublets (Fig. 2C, Supplementary Fig. 4). No larger aggregates were observed. The doublets that were not recognized as such in the SSC-H vs. SSC-A plot, since this area was excluded in the P1 population (Supplementary Fig. 2), might in some cases represent the ´smallest sortable units´ for cells in late stages of cell division or otherwise tightly interacting cells.

The *sar*A-dependent fluorescence reporter signal has primarily served as a simple tool for detecting cells. However, since we observed a high level of heterogeneity in single-cell derived growth post-sorting (Fig. 2A), we wondered if the reporter signal strength at the time of sorting (and thus either the transcriptional status of the cell or its plasmid copy number) would correlate with the growth patterns. To investigate this, we sorted single events differing in GFP fluorescence intensities: low, intermediate and high. When outgrown in the presence of antibiotic selection, cultures derived from GFP^high^ cells had marginally (although in part statistically significant) lower lag phases* (Fig. 2D-ii) and generation times (Fig. 2D-i) compared to cultures derived from cells with lower GFP signals (Supplementary Data 1). We suspected that this outcome might be affected by plasmid copy number, which we determined by qPCR to be on average higher in sorted GFP^high^ compared to GFP^low^ subpopulations (Fig. 2E). We therefore decided to compare the outgrowth kinetics of sorted subpopulations in the presence and absence of antibiotic selection. While in the presence of antibiotic selection, high plasmid copies numbers, may provide a growth advantage, this advantage would be lost in the absence of antibiotics, where plasmid carriage might rather inflict a fitness cost. For all sorted subpopulations the median generation times and lag phase* were lower in the absence compared to the presence of antibiotic (Fig. 2D, F, G, Supplementary Data 1). This difference in lag phase and generation time between the TSB and TSBCm was more pronounced for GFP^int^ and GFP^high^ subpopulation than for GFP^low^ (Fig. 2G), suggesting that higher plasmid copy numbers in the former could provide a growth advantage in the presence of antibiotics. In fact, whereas 80% of sorted GFP^low^ cells gave rise to cultures in antibiotic-free media, this percentage dropped to 50% in the presence of antibiotic. In comparison, the percent recovery rates for the GFP^int^ and GFP^high^ cells were between 84 and 89% in the presence and absence of antibiotics. The inability of a significant fraction of single GFP^low^ cells to grow in the presence of antibiotic selection could for example be explained by too low copy numbers in individual cells resulting in insufficient expression of the chloramphenicol resistance gene. Complete plasmid loss in some cells cannot be ruled out either, even though the plasmid was stable in bulk culture assays over one growth cycle in TSB without antibiotic selection (Supplementary Fig. 3F). Interestingly, in the absence of antibiotic selection, all sorted subpopulation cultures displayed a bimodal lag phase* distribution (Fig. 2F), clearly distinguishing subpopulations with lag phase * <13 h (´low lag´) and those with a longer lag phase* >13 h (´high lag´) (Fig. 2F-ii, 2F-iii). Of note, cells with a higher GFP signal at the time of sorting included higher numbers of ´low lag´ cells (% ´low lag´: GFP^low^: 40.2%, GFP^int^: 62.2%, GFP^high^: 73.7%, N: 102- 114) (Fig. 2F-iv). If this system was confounded by plasmid copy numbers and related fitness defects, it could be expected that GFP^high^ cells show the worst growth.

The reduced lag phase* times observed for cultures derived from GFP^high^ cells is the opposite outcome of what one might expect if the higher GFP signal resulted from confounding effects of higher plasmid copy numbers in this subpopulation of cells, as any associated fitness cost would rather negatively affect growth in the absence of antibiotic selection. Our data therefore rather suggest that it is indeed the *sar*A promoter activity status that is associated with earlier outgrowth of cells and serves as a proof-of-principle demonstration of our single-cell derived growth analysis.

### Single cell-derived growth analysis detects bimodal growth phenotypes independent of colony microanatomy

**Fig. 3:**
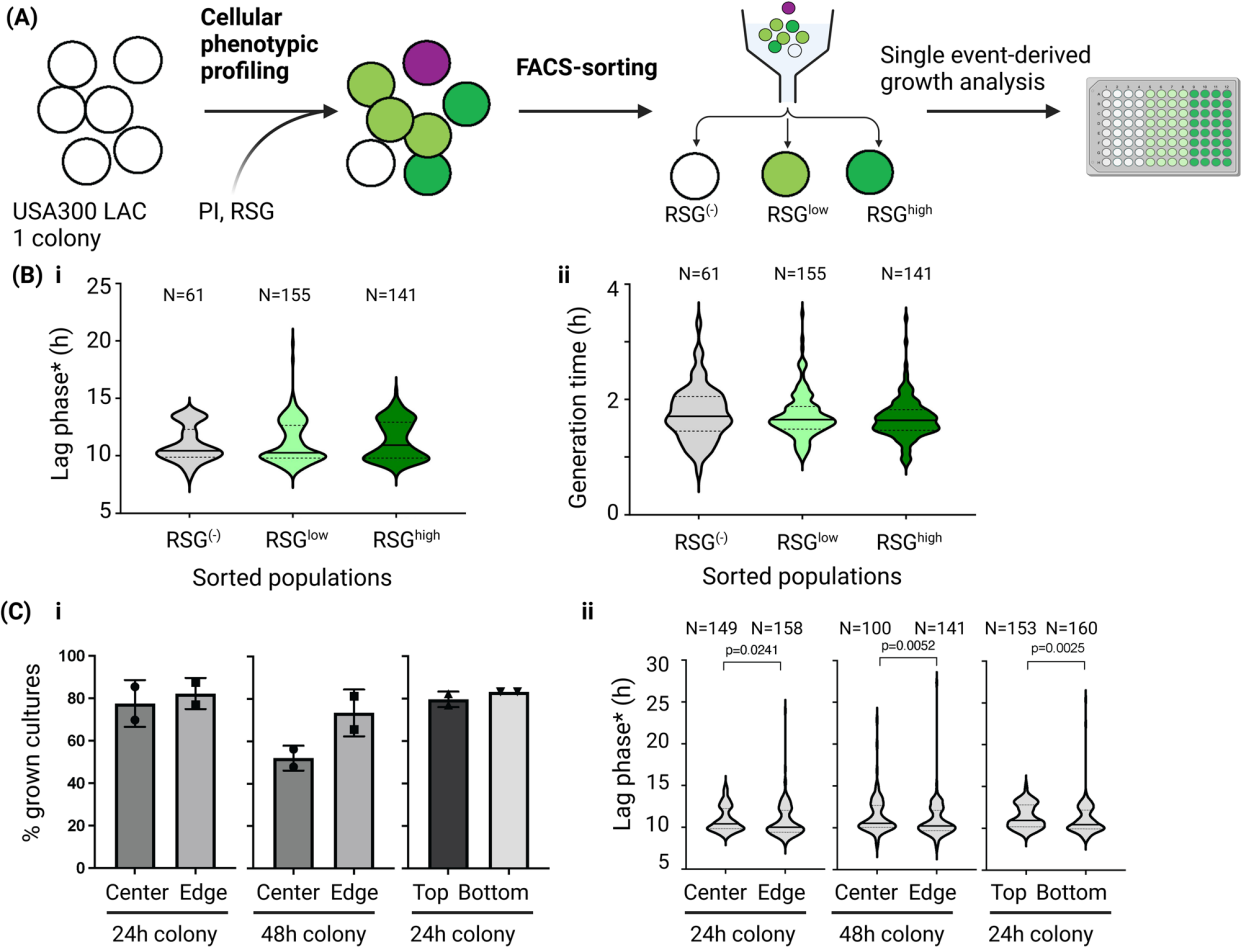
Heterogeneity of cellular growth phenotypes in *S. aureus* USA300 LAC (WT) colonies. **A** Schematic overview of the experimental setup. Cells from a single WT colony (pre-sorting culture) were stained with RSG and PI. RSG^(–)^, RSG^low^ and RSG^high^ subpopulations were sorted for single-event derived growth analysis. Gating scheme is presented in in Supplementary Fig. 2C,D. **B** RSG phenotype pooled from 3 independent pre-sorting cultures, (i) Lag phase* (h) and (ii) generation time (h) of single-cell derived cultures derived from RSG^(–)^, RSG^low^ and RSG^high^ subpopulations in TSB liquid media. Data were not significantly different. **C** Single-cell derived growth performance of unlabelled WT cells harvested from different locations of a colony derived from 2 independent pre-sorting cultures. Bacteria were harvested from either center and edge or top and bottom of a colony grown for 24 or 48 h, sorted from SSC^low-P1^-PI^-^ population for single events and were subjected to single-cell derived growth analysis in TSB liquid media, (i) Percentage of positive cultures grown for 48 h post single event sorting. The bars represent average ± SD that were derived from 96 sorted events each. Data were not significantly different, (ii) Violin plot showing lag phase (h) of single-event derived growth in TSB liquid media. Statistical analysis in **B** was performed by Kruskal–Wallis test in combination with Dunn´s multiple comparison test and in **C** with two-tailed Mann–Whitney test. N represents sample size in violin plots. Only significant differences are shown and represented as *P*-values. Cross lines in all violin plots represent median value. Raw data and calculations are available in Supplementary Data 1. The figure was created with BioRender.

To test growth heterogeneity in colonies without the confounding factors associated with the plasmid-based reporter strain, we decided to study wildtype (WT) cells from single colonies of bacteria of the MRSA strain USA300 LAC. We labeled cells with a live-dead stain combination that included PI and the metabolic reporter dye Redox Sensor Green^TM^ (RSG) (Fig. 3A), which is activated by reductases in the respiratory chain. In contrast to the sharp fluorescence signal achieved with the *sar*A-GFP reporter strain, the RSG signal showed a wide distribution that was dose-dependent and revealed a minor RSG negative (RSG^(–)^) population (Supplementary Figs. 2D, 5). Cells from RSG^(–)^, and cells with low or high RSG-signal (RSG^low^, RSG^high,^, Supplementary Fig. 2D) were sorted for single event-derived growth analysis in liquid media. The first notable observation was that 31% of RSG^(–)^ events led to growth (Supplementary Data 1) suggesting they are live cells, despite their inability to stain with the metabolic dye. One possible explanation could be limited permeability of the probe in this population. Next, when comparing the growth kinetics of different single-cell derived cultures, we observed large cell-to-cell heterogeneity in lag phase and a bimodal distribution with peaks at ≈10 h and 14 h, respectively (Fig. 3B-i). This observation was similar to that seen in the *sar*A-GFP reporter strain grown without antibiotic selection (Fig. 2F-ii). In contrast, generation time was highly similar for all single cell-derived cultures (Fig. 3B-ii). Of note, the staining procedure itself did not have any untoward impact on cell physiology, and single-cell derived cultures from unstained, PI-, RSG-, or double-stained cell populations had identical lag phase* distributions (Supplementary Fig. 3G). Our data thus suggest the presence of two physiologically distinct subpopulations in the cell population pre-sorting. Since cultures derived from RSG^(–)^, RSG^low^ and RSG^high^ cells all showed similar bimodal distribution patterns, we conclude that the downstream growth performance is independent of the metabolic/redox-status reported by RSG during sorting (Fig. 3B-i, ii).

We wondered if this bimodality could be explained by the microanatomy of the colony, i.e., that cells in the center vs. edge might differ in cell age and lag phase, or whether direct nutrient availability influences growth kinetics of cells in the top or bottom part of the colony. We therefore harvested cells from different locations and compared their lag phase and growth kinetics. We found that cultures harvested from the center of the colony included a higher percentage of events that did not result in downstream growth post sorting, suggesting the presence of either dead or VBNC cells (Fig. 3C-i). Cells from the bottom and edge had lower lag phases than cells from the center or top, respectively (Fig. 3C-ii). However, the bimodality was observed throughout all four conditions, and the location of the cells in the colony did not influence their outgrowth kinetic, suggesting that this level of heterogeneity is not primarily dictated by the location and thus microenvironment of the cells.

### *Phenotypic backtracing* connects cell fate analysis of dormant subpopulations with single-cell phenotypic profiles

**Fig. 4:**
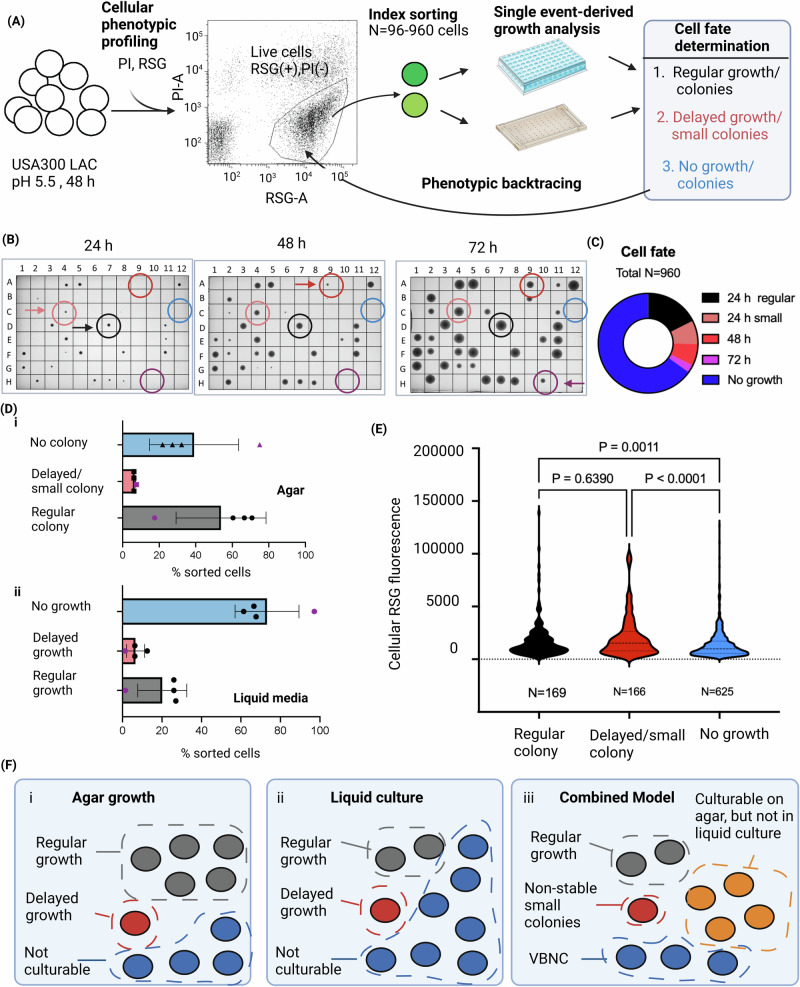
Cellular phenotypic profiling and backtracing of dormant *S. aureus* subpopulations. **A** Schematic overview illustrating the experimental setup. After cultivation in media with low pH for 48 h, *S. aureus* USA300 LAC cells were labeled with RSG (for live cells) and PI (for dead cells), followed by flow cytomety analysis. Using an index sorting approach that enables phenotypic backtracing, live cells (PI^–^) were subsequently sorted for single-cell derived growth analysis on agar and in liquid media. **B** Exemplary images of the same representative agar plate showing colony growth at (i) 24 h (ii) 48 h, (iii) 72 h after sorting of 96 individual events sorted as single cells while the bottom image displays colony growth after 48 h. Different cell fates are highlighted with colored arrowheads: Normal (gray), delayed/small colonies at 24 h (light red), colonies appearing at 48 h (dark red), colonies appearing at 72 h (purple), and no growth (blue). **C** Overview of the distribution of cell fates determined for 960 sorted events. **D** Distribution of single-cell derived growth phenotypes (regular growth, delayed growth and no growth after 24 h) on (i) solid media and (ii) liquid media. Bars show means ± s.d. of *n* = 4 independent pre-sorting culture replicates (derived from 2 experiments). For each pre-sorting culture the same amount of cells was sorted on solid and liquid media for comparative growth analysis. Black dots correspond to the biological replicates derived from an experiment where *N* = 96 events were sorted per condition, purple dots from a separate experiment with *N* = 960. **E** Violin plots displaying the RSG-A fluorescence intensity of ´backtraced´ index-sorted cells after growth analysis on TSA (*N* = 960). Events are organized according to cell fates including regular colony size, delayed growth (i.e., all events giving rise to small colonies at 24 h or colonies that appear at 48 h or 72), and no growth within 72. Statistical significance testing was performed by Kruskal–Wallis test in combination with Dunn´s multiple comparisons test. **F** A model showing the composition of the low pH-elicited *S. aureus* cell populations based on the observed growth phenotypes assessed in (**D**). (i) Average composition of subpopulations with respect to cultivability of single cells on agar and (ii) in liquid culture (TSB), each including regularly culturable cells (gray), cells displaying delayed growth (red) and non-culturable cells (blue). One indicated cell is representative of ca. 10% of the cell population. (iii) A combined model taking into account growth analysis on both media, highlighting the existence of a subpopulation of differentially cultivable single cells that give rise to colonies on agar, but fail to yield detectable growth in liquid media (orange). Cells displaying delayed growth may represent non-stable small colonies and non-culturable cells as VBNCs. The figure was created with BioRender. Raw data and calculations are available in Supplementary Data 2.

Having dissected heterogenous growth phenotypes in colonies, we sought to address dormant, i.e., non-stable small colony phenotypes that can be elicited by prolonged growth at low pH^11,43^ (Fig. 4A). Cells were grown in DMEM adjusted to pH 5.5 for 48 h prior to labeling with RSG and PI and FACS-analysis. RSG^(^^+^^)^, PI^(–)^ (i.e., putatively live) cells were then sorted for downstream single-cell derived analysis and colony formation was observed initially for 48 (Supplementary Data 2) (*N* = 3 entirely independent culture replicates pre-sorting and 96 cells sorted per sample). Monitoring colony formation for 48 h revealed that 66.5% of sorted events gave rise to ´regular´ colonies, whereas 6.25% of events gave rise to small/delayed colonies. In other words, 8.6% of all formed colonies were small which concurs with previous observations by Huemer et al. ^11^. In 27.25 ± 5% of sorted events no colony appeared within 48 h (Supplementary Data 2). These cells might be in a deeper state of dormancy or VBNC-state^52,53^. Since we sporadically observed colonies appearing as late as 72 h after sorting we performed another experiment at tenfold higher scale sorting and analyzing 960 cells from a single starting culture to increase the likelihood of detecting such lower frequency events. In this experiment overall recovery was lower, with 17.6% of sorted events growing regularly (colonies >1 mm after 24 h), 7.5% yielding small colonies after 24 h, 7.1% yielding detectable colonies after 48 h and 2.6% after 72 h (Fig. 4B, C, Supplementary Data 2B, C). Our results show that the population of events that does not give rise to colonies by 24 or even 48 h indeed contains viable cells that become ´culturable´ later.

Dormant growth variants have commonly been observed on solid media but are impossible to detect with traditional bulk-based liquid culture techniques. FACS-sorting, however, allows for single cell-derived outgrowth studies in liquid culture and revealed that the percentage of sorted events that were not able to yield detectable growth was 34 ± 8% (*N* = 4) higher in liquid media compared to events that failed to grow on solid media (Fig. 4D, Supplementary Data 2D, E). Thus, not all single cells that give rise to colonies on agar are able to grow in liquid media (see model in Fig. 4F).

One common limitation in the field of persisters/non-stable small colonies is that their phenotype can only be inferred retrospectively once cells have reverted from dormancy into a proliferating state. To overcome this limitation, we developed *phenotypic backtracing* (Fig. 4A). Phenotypic backtracing connects the fate of a cell post-sorting (e.g., after determining whether the cell was dormant or not) with a phenotypic characterization of a cell pre-sorting (e.g., while a cell *is* dormant). Here, we classified the growth phenotypes displayed by cells post-sorting into three cell fate categories: (i) regular growth, (ii) delayed growth (i.e., appeared as small colonies <1 mm diameter at 24 h or appeared at 48 or 72 h), (iii) no growth. For each sorted event we could *trace back* the founder cell in the FACS-plot, thus connecting a cell´s fate with its RSG fluorescence at the time of sorting (Fig. 4C, E, overview of colony growth on all plates in Supplementary Fig. 6). Backtracing of RSG fluorescence of *N* = 960 sorted events analyzed for growth on agar revealed that non-culturable cells had significantly lower RSG fluorescence compared to cells with regular (1.3-fold lower median RSG fluorescence) and delayed growth (1.6-fold lower median RSG fluorescence) (Fig. 4E, Supplementary Data 2). On the other hand, cells that showed delayed growth did not show differences in the RSG signal compared to regularly growing cells. Our data suggest that CPP with RSG may help discriminate VBNCs from culturable cells, but does not distinguish regularly growing cells from presumable non-stable small colony/persister populations that show a delayed growth phenotype.

### Cellular phenotypic profiling denotes isogenic subpopulations with reduced vancomycin susceptibility in vancomycin-intermediate susceptible *S. aureus* (VISA)

**Fig. 5:**
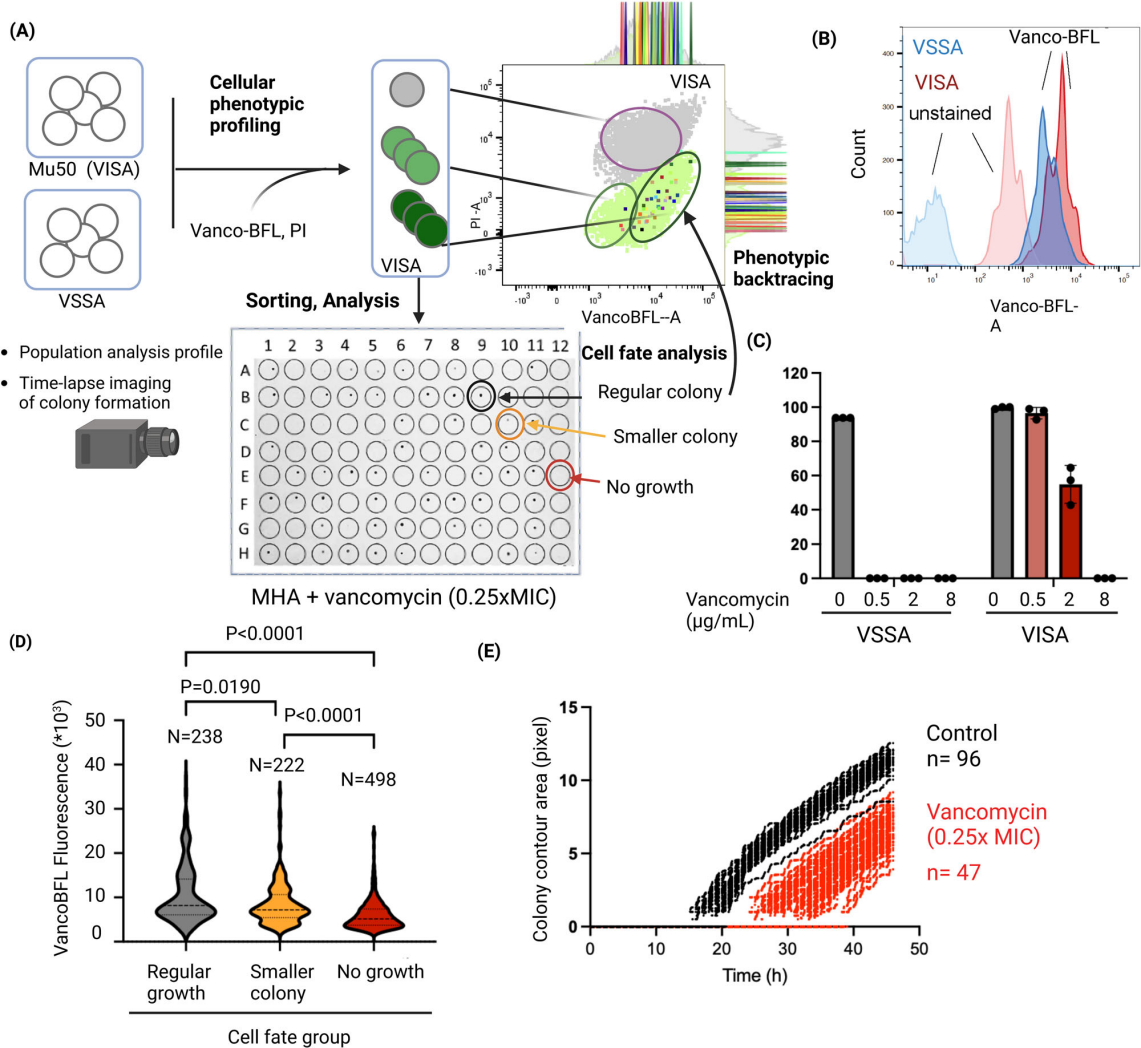
CPPT detects heterogeneity in vancomycin resistance in a VISA strain. **A** Schematic overview of the CPPT setup for comparative analysis of VISA strain Mu50 and a VSSA control. Cells were labelled with Vancomycin-BFL and PI. PI^(+)^-cells are depicted in gray, PI^(–)^-cells with different degrees of Vanco-BFL-labeling are indicated in light and dark green. PI^(–)^-cells were sorted onto Mueller-Hinton Agar (MHA) supplemented with different concentrations of vancomycin and colony formation was observed at different time points and for individual plates by time-lapse imaging. For VISA cells growth on a 0.25× MIC plate was used for cell fate determination (regular colony, delayed, smaller colony or no colony) prior to phenotypic backtracing of the ancestral phenotypic signatures in the flow cytometry dot plot. The exemplary dot plot shows all recorded Vanco-BFL^(^^+)^, PI^(–)^ events in light green, Vanco-BFL^(+)^, PI^(+)^-events in grey. The individually colored dots correspond to backtraced, sorted cells that were determined to belong the ´regular colony´ cell fate category. **B** Flow cytometry histogram showing the fluorescence signal in the Vanco-BFL-A channel of unstained and Vanco-BFL-labelled VSSA (blue) and VISA cells (red)´. **C** Percentage of sorted events that gave rise to colonies on agar plates with different concentrations of vancomycin. **D** Violin plot showing the cellular Vanco-BFL signal intensity of VISA cells for the different cell fate groups determined after growth on 0.25xMIC vancomycin. The graph shows the results of *N* = 960 sorted events from a single pre-sorting culture and is representative for 4 biological replicates. Statistical significance testing was performed by Kruskal–Wallis test in combination with Dunn´s multiple comparisons test. **E** Colony growth dynamics of sorted VISA cells in the presence (0.25xMIC) and absence of vancomycin as analyzed by time-lapse imaging analysis (compare Supplementary Movies 1, 2). Growth (*Y*-axis) was measured as contour size on the edge of the colony and is plotted against cultivation time. Raw data and calculations are available in Supplementary Data 2. The figure was created with BioRender.

Whereas chemical probes for dormant phenotypes remain to be developed, some probes whose labeling profiles may correlate with the susceptibility to certain antibiotics exist. Zhang et al. demonstrated that cellular labeling profiles of *S. aureus* strains with fluorescent analogs of the glycopeptide antibiotic vancomycin were dependent on molecular characteristics of vancomycin resistance^54^. Vancomycin targets D-Ala-D-Ala in peptidoglycan precursors peptides. Vancomycin-resistant *S. aureus* (VRSA) strains produce a modified peptidoglycan peptide sidechain with low affinity for binding vancomycin and thus the fluorescent vancomycin analog. This results in reduced cellular labeling with fluorescent vancomycin analogs in VRSA compared to the susceptible strains (VSSA)^54^. In contrast, VISA strains, for which resistance is associated with decreased autolysis, increased cell wall thickening and putatively production of decoy targets^55–58^, revealed increased labeling of vancomycin probes compared to VSSA^54^. Since VISA and heteroresistant VISA (hVISA) commonly display heterogeneous resistance patterns^59,60^, we asked if fluorescent vancomycin probes could be used in CPPT to expose subpopulations with different susceptibility to vancomycin^59^ (Fig. 5A).

We compared the labeling profiles of the VISA strain Mu50^61^ and a VSSA control with the commercially available fluorescent probe Vancomycin-BODIPY FL (Vanco-BFL). Consistent with the observation by Zhang et al. ^54^, we found that VISA cells labelled more strongly (Fig. 5B). Interestingly, also in the absence of probe, VISA cells showed increased autofluorescence in the fluorescein channel, which may be attributed to its increased cell wall thickness^56,57^. The flow cytometry profile further indicated subpopulations with different Vanco-BFL signal in the VISA cell population. To determine if subpopulations of the VISA strain differ in their antibiotic susceptibility we sorted single cells onto agar with different concentrations of vancomycin, thereby establishing a traceable variation of the population analysis profile test which is commonly used to detect heteroresistance^58^. Whereas cells grew uniformly at 0.0625xMIC vancomycin and no colonies formed at 1xMIC (=8 μg/mL), at 0.25xMIC 55% of the single cells grew to colonies supporting the existence of subpopulations with different levels of susceptibility to vancomycin (Fig. 5A, C, Supplementary Fig. 7). To test if this observed heterogeneity in vancomycin-susceptibility was correlated with the cellular Vanco-BFL labeling profile, we implemented *phenotypic backtracing*. Sorted events were assigned into three cell fate categories depending on their ability to grow and the size of the resulting colony after 48 h. Colonies with a diameter >1 mm were classified as ´regular´, whereas colonies with a diameter <1 mm were categorized as ´smaller colonies´. Phenotypic backtracing analysis with up to *N* = 960 events from a pre-sorting culture revealed that cells displaying regular growth localized to a specific area of the Vanco-BFL– PI plot and showed significantly higher Vanco-BFL signals compared to cells from the other cell fate groups (≈1.6-fold higher median Vanco-BFL fluorescence values compared to cells unable to grow and ≈1.1-fold higher values compared to cells that give rise to smaller colonies, Fig. 5A, D; Supplementary Fig. 8, Supplementary Data 2). Thus, Vanco-BFL discriminates cellular subpopulations with different susceptibility to vancomycin and VISA cells that display higher Vanco-BFL labeling are more likely to survive vancomycin treatment. Since colonies recovered in the presence of 0.25xMIC vancomycin had a reduced size, we implemented an in-house built solution for automated time-lapse imaging of colony formation post-sorting, adapted from ColTapp^62^, to determine if (a) surviving cells were sufficiently pre-adapted to grow in the presence of vancomycin but do so at a slower rate (explaining the small colony size) or (b) if cells needed to undergo additional steps of functional adaptation, leading to a delayed onset of growth. The experiment revealed that in the presence of vancomycin colonies became detectable on average 12–14 h later than in the absence of antibiotic (Fig. 5E, Supplementary Movies 1, 2), but once detectable, colonies grew at similar rates in the presence and absence of vancomycin. Collectively, our results indicate that the cellular trait causing increased labeling with Vanco-BFL in a subpopulation of cells is a necessary, but not sufficient precondition to survive antibiotic exposure, and that the ability to grow in the presence of vancomycin required additional functional adaptations inducing a growth delay. This finding concurs with reports on changes in metabolism and cell wall biosynthesis gene expression in VISA strains upon vancomycin exposure^63^. It remains to be determined if differences in cell wall structure, such as absence of glutamine amidation and increased cell wall thickness which were reported as general characteristics of Mu50^57^ are also the molecular basis for the observed heterogeneity in Vanco-BFL labeling and vancomycin-susceptibility.

## Discussion

Understanding cell individuality is an important precondition to understand cooperative behavior in bacterial populations. When individual cells are morphologically identical, which is the default scenario for most isogenic populations, differentiating functionally different cell is a formidable challenge. In recent years several technical advances have been made. Single-cell sequencing approaches, for example, provide in-depth and high-throughput (HT) information about the transcriptional status of cells^64,65^, but their use requires the destruction of cells, preventing further functional analysis. On the other hand, microfluidic studies have emerged as the gold standard for visualizing specific phenotypic traits using fluorescent markers while allowing for a microscopy-based assessment of replication and antibiotic susceptibility in real-time^35,36,39^. Yet, the general throughput is limited, functional analysis largely restricted to microscopy and analysis of bacteria whose growth requires cell division events in multiple planes is challenging. We are convinced that our flow cytometry-based CPPT-platform provides valuable, complementary information for deconstructing cellular phenotypic heterogeneity in bacterial populations. Whereas microfluidics studies directly track single cells and their replication microscopically, CPPT differentiates and separates single-cells, but the growth kinetics are detected in more traditional growth assays with the distinction that they are single cell-derived. In the future, this observation gap may be closed by combining cell sorting with downstream real-time-microscopy in microfluidic systems.

In combination with simple single-cell derived growth assays, CPPT has exposed previously unidentified heterogeneity-related phenomena, such as the bimodal distribution of lag phases not related to colony topography, or subpopulation of single cells that are culturable on agar, but not in liquid culture.

We observed bimodal lag phase distribution both for colony-derived cells of a WT strain as well as the plasmid-based *sar*A-GFP reporter strain when outgrown in the absence of antibiotic selection. Our results with the *sar*A-reporter strain demonstrated that cells with a higher GFP signal were more likely to display a ´low lag´ phenotype, whereas GFP^low^ were more likely to display a growth delay (´high lag´ phenotype). Whether this phenotype is related to the specific function of *sar*A as a gene regulator or reflects the general transcriptional status of the cells remains to be determined. Of note, in the presence of antibiotic selection, the single cell-derived growth profile was not bimodal, but multimodal. We hypothesize that the underlying bimodality was occluded by single cell variations in plasmid copy number and corresponding differences in antibiotic resistance gene expression and fitness to grow in the presence of antibiotic selection, which we expect to further diversify the single-cell growth distribution. These data indicate that caution needs to be taken when performing CPPT-based growth assays using plasmid-based reporter strains and we discourage outgrowth analysis in the presence of antibiotic selection.

Our results from the analysis of WT colonies, instead showed that neither the metabolic status reported by RSG, nor the location of the cell in the original colony affected the bimodality. A systematic analysis of colonies of different ages or the comparison of mutant strain libraries might reveal additional insights into the dynamics of these subpopulations and the underlying molecular mechanisms. Another very interesting subpopulation phenotype revealed by CPPT are single cells that are culturable on agar, but not in liquid culture (see model in Fig. 4F). Since this different cultivability of single cells on agar vs liquid culture was observed with cells grown at low pH, we assume that it is a characteristic of stressed cells, and it may be connected to known dormancy phenotypes such as VBNCs and persisters which are triggered under stress. We hypothesize that the physical contact with agar surface might somehow help stressed cells to recover and start replication, whereas planktonic single cells fail to do so, but the molecular reasons underlying this phenomenon remain to be determined. Our findings represent a new nuance in bacterial cultivability and adds another level of complexity and additional fuel to the ongoing debate of when cells are viable, culturable, dormant, or dead^25–28^. Our data also suggest that caution must be taken when comparing studies addressing dormancy and cultivability in agar-based systems, compared to e.g., microfluidics-based studies performed in a (confined) liquid environment. The mechanisms underlying these newly described phenotypic variants remain to be determined. Yet, these results illustrate the power of CPPT to uncover fundamental new aspects of bacterial physiology and ecology. The flow cytometry-based platform is tunable to operate at the single-cell level, as in this work, but can also be used with gating to sort subpopulations with a specific labeling profile for diverse downstream assays, such as ´omics´ studies or infection or virulence-related assays.

CPPT also provides new opportunities for dissecting known cellular subpopulations. For persister research, one experimental dilemma is that the phenotype can only be assigned retrospectively after cells have undergone a phenotypic reversal to a replicating state. Our platform provides a potential solution that combines determination of the cell fate (e.g., to confirm dormancy by the ability of cells to grow after an increased lag), with backtracing to cellular phenotypic profiles determined by flow cytometry. This strategy allows for broad-scale phenotypic characterization of dormant cells using diverse combinations of fluorescent chemical probes that could enable identification of markers for the direct detection and enrichment of dormant cells. Here, we tested if RSG, a commonly used metabolic marker of cellular reductase activity, would be associated with dormancy. We observed that the RSG signal of readily growing cells was not different from that of cells showing delayed growth, which presumably represent non-stable small colonies/persisters. This finding concurs with previous observations that although *S. aureus* persisters are associated with low ATP levels and reduced metabolic activity^42,66^, metabolism and transcription are not completely abolished^11,43^. We did observe however that cells that were not culturable on agar (despite being RSG-positive and PI-negative) showed reduced RSG signals compared to growing cells. Even though the RSG signal distribution of non-culturable and culturable cells were not cleanly separated, but overlapping, this finding suggests that RSG signal might help to distinguish ´deeply dormant´ VBNCs from ´less dormant´ persister cells.

That labeling with chemical probes can in fact be used to differentiate clinically relevant subpopulations is supported by our finding that Vanco-BFL marks a subpopulation of VISA cells with increased vancomycin-resistance. This clear distinction is remarkable since strain Mu50 is considered more homogenous in its resistance profile than hVISA strains^3^, for which even more pronounced differences in labeling may be expected. Cellular labeling with fluorescent vancomyin-conjugates has been shown previously to characterize strain-specific differences in vancomycin susceptibility correspondint to different resistance mechanisms^54^. Our study demonstrates that that the utility of these probes as a biomarker can be expanded to the detection of subpopulation-based differences in vancomycin-resistance, which may have important implications for the detection of heteroresistance e.g., in clinical samples.

Whereas the flow cytometry analysis is high-throughput and can easily measure 1e^6^ or 1e^7^ cells, the functional analysis and cell fate determination of sorted cells has a lower throughput. Here, we included single-cell growth analysis in liquid culture and on agar. For single-cell derived liquid culture analysis, the maximum throughput is dictated by the capacity of the plate reader (which is up to 10 plates for the instrument used in this study, allowing for simultaneous assessment 960 events in 96-well plates). For growth analysis on agar, there is no such limit set by the instrumentation (unless time-lapse imaging of colony growth is performed, which is limited by the capacity of the imaging chamber used). Here we sorted out up to 960 cells on agar per experiment, which was high enough for robust detection and statistical analysis of the cell fates of interest occurring at frequencies of >1%. Of note, in some clinical samples of difficult-to-treat *S. aureus* infection samples non-stable small colonies can be observed at similar or even higher frequencies^11,13^, suggesting the compatibility of CPPT with the analysis of such samples. Whereas it is possible to further increase the scale of the experiments to reliably detect less frequent phenotypes if required by the aims of the study, in some samples spontaneous persister cells or heteroresistant cells can occur at much lower frequencies of 1e^–4^ or 1e^–5^. Such rare events will be challenging to detect by CPPT if cells are randomly sorted from the entire cell population. Such rare events will however be registered in sufficient numbers during flow cytometry analysis (CPP) and could be enriched for by gating on subpopulations characterized by fluorescent probe labeling patterns. To identify probe combinations that might positively or negatively stain target cells of interest, CPPT can be used under model conditions where the cell types of interest occur at higher frequencies, such as the low pH conditions used in this study and others^11^ to trigger non-stable small colonies, or heteroresistant cells that are selected for by antibiotic treatment.

Chemical Biology is providing an ever increasing toolset of next-generation physiology probes that report on diverse phenotypic traits from cellular permeability, specific biomolecular uptake pathways, replication, metabolic activity or the distribution and activity of specific molecular targets or groups in living bacterial cells (as reviewed in refs. ^34,67,68^) and are exploitable for CPP. The use of exogenous fluorescent probes makes CPP highly compatible for applications in clinical samples^13^, allowing for direct detection and characterization of unstable phenotypic variants that would revert upon cultivation. We believe persisters and heteroresistant cells are only the most easily detected tip-of-the-iceberg of distinct subpopulation phenotypes in isogenic bacterial pathogen populations. A systematic implementation of CPPT will help uncover further clinically relevant subpopulation phenotypes, understand their ecological role and interplay with other cells in the populations, and enable development of strategies to detect, target, or manipulate subpopulation phenotypes to improve the clinical outcome of bacterial infection management and antimicrobial treatment.

## Methods

### Bacterial strains and growth conditions

*Staphylococcus aureus* (*S. aureus*) strains analyzed in this work are summarized in Table 1. Wildtype (WT) strains were routinely cultivated in Tryptic Soy Broth (TSB) liquid media or Tryptic Soy Agar (TSA) solid media at 37 °C unless stated otherwise. The fluorescent *sar*A-GFP reporter strain transformed with plasmid pCM29 (harboring *sarA*-GFP reporter; courtesy of Dr. Alexander Horswill)^48^ was grown on TSB or TSA supplemented with 10 µg/ml chloramphenicol (TSBcm or TSAcm).

**Table 1:** Bacterial strains used in this study

Strain	Description	Reference or Source
*S. aureus* LAC	Community-acquired MRSA clone from the USA300 lineage, isolated from Los Angeles County (LAC). This strain is referred as WT strain	^32,80^
*S. aureus* ATCC 29213	*S. aureus* subsp. *aureus* Rosenbach, VSSA, Wild type Strain Rosenbach, Performance Standard for Antimicrobial Susceptibility Testing	ATCC
*S. aureus* ATCC 700699	Strain Mu50, VISA, Wild type	^59^ ATCC
*S. aureus* USA300 LAC/pCM29	*S. aureus* USA300 LAC strain carrying *sar*A-GFP reporter plasmid pCM29(Chloramphanicol resistance). This strain is referred as *sa*rA-GFP reporter strain. See Supplementary Fig. 1 for plasmid map and Supplementary Data 5 for plasmid sequence.	^48^, Dr. Alexander Horswill

### General flow cytometry and cell sorting

All samples in this research were analyzed by flow cytometry and sorting instrument BD FACSAria III instrument (BD Biosciences). The instrument was configured and pre-calibrated with BD® CS&T beads according to the manufacturer’s protocols to conduct quality assessments of the instrument’s optical, electronic, and fluidic systems, as well as facilitate the calibration of fluorescence compensation. Before sorting, BD FACS™ Accudrop Beads were used to ensure proper drop formation and sort accuracy during the flow cytometry and sorting process. A neutral density (ND) filter 1.0 was used to regulate the laser power and optimize signal-to-noise ratio during data acquisition. Importantly, 70 μm nozzle with a sheath pressure of 70 psi was used for sorting to achieve optimal separation of cells and minimize clogging of the instrument. All other parameters such as FSC (Forward scatter), SSC (Side scatter), fluorescence channels and their voltage, threshold were adjusted as needed based on experiments and optimization. Flow rates and sort purity during sorting were maintained at a consistent and optimal level to ensure efficient and accurate separation of cells and adjusted based on sample and sorting objective. Instrument settings were set, and data acquisition was performed using BD FACSDiva software V 9.0 and FlowJo software (V 10.8.1) were used for data analysis.

### Imaging flow cytometry

Imaging flow cytometry was performed on an ImageStreamX MkII (Amnis). The detailed procedures are described under specific experimental procedures for proof-of-principle studies with fluorescent reporter strain below.

### Phenotypic backtracing

We employed the phenotypic backtracing method to analyze the observed ancestral cell functions that translates into functional traits exhibited by bacterial colonies at single-cell resolution. In this pipeline, backtracing of cell function was implemented through indexing of individual cell, which are to be sorted. Index sorting represents a FACS sorting mode that enables the isolation of individual bacterial cells while allowing for a retrospective assessment of all fluorescence and scatter parameters associated with each cell. BD FACSAria III instrument was used for index sorting. The indexing parameter were set to single-cell precision with target event 1 and the sorting layout to 96-well plate. Single-cell precision does not allow interrogated drop to be sorted if 2 target cells are present. Index data was further analyzed with FlowJo software (V 10.8.1) and its indexSort package. This package retrieve index-sorted data from fcs data files and visually explore for downstream analysis.

### Single- or multiple event derived growth kinetics in Broth media

Single or multiple flow cytometry events (i.e., cells) were directly sorted into individual wells of a 96-well plate (353072, Falcon) containing 200 µl of either TSB (for WT strain) or TSBcm (for *sar*A-GFP reporter strain) based on experimental condition. Plates were then sealed with Breathe-Easy sealing membrane (Z380059, Merck) and incubated in the Biospa 8 (Biotek) at 37 °C and 80% humidity. Absorbance at 600 nm (A600) was measured every 30 min for a period of 48 h using Synergy H1 (Biotek) plate reader. Prior to each read, plates were shaken orbitally at low speed for 10 s following by a 5 s pause. Biospa 8 and Synergy H1 were operated via Biospa on Demand and Gen 5.3.10 software, respectively. Data were exported to an excel file (.xlsx) using Gen 5.3.10 software. Details about modeling different phases of bacterial growth is presented in Supplementary Data 4. Data files were curated such that they contained time in 00:00:00 format (column A), temperature (column B) and A600 values from wells A1 to H12 in subsequent columns. Headers and all other information were removed from curated xlsx files, which were saved with an extension of _A.xlsx (e.g., FILE NAME_A.xlsx). An example of curated file is presented in Supplementary Data 4. Exported data was further analyzed using MATLAB script (see code availability), which generates growth rate (h^–1^), lag phase* duration (h) and onset of stationary phase (h) and generation time (h) was calculated as LN (2)/growth rate. Since our method does not measure the true lag phase, which can be measured through microscopic observation of individual cells, we are reporting a related parameter that we designated ´lag phase*´. With ´lag phase*´, we are referring to the time duration which a freshly inoculated culture required to register a significant measurable increase in absorbance (A 600) above media background in the plate reader assay.

### Growth kinetics on agar media

Single or multiple flow cytometry events were directly sorted in a single well plate (internal dimensions 118.63 × 82.13 mm; 734-2977, VWR) containing ~45 ml of either TSA (for WT strain) or TSAcm (for *sar*A-GFP reporter strain), and Mueller-Hinton agar (MHA) with or without antibiotic (Vancomycin). Single well plates were sorted using single cell precision mode by the adjusted 96-well sorting layouts in FACSDiva software. Following sorting, plates were placed inside of in house built real time colony tracking imaging platform. The platform comprises metal box with preinstalled cannon eos M200 camera with central temperature control condition, which at 37 °C. Images from agar plate were acquired every 5 min interval for 24 h (total of 289 frames) or 48 h (578 frames) and converted into time-lapse single video file.

### Time-lapse imaging of colony growth

The resulting video files of single cell growth on agar plates were analyzed with in house-built software for tracking colony growth dynamics in real-time. The process of colony detection involves a series of image analysis operations, starting with a grayscale image. Local adaptive thresholding is applied to create a binary image, followed by artifact removal and the elimination of unwanted objects based on size criteria. Overlapping colonies are separated using watershed segmentation. Subsequently, this code employs circular filtering and contrast enhancement on isolated object images. To address false positive detections, sequential quality control functions and Mahalanobis Distance^69^ estimation are used to exclude them from further analysis. Overall, the process includes multiple steps, such as thresholding, artifact removal, watershed segmentation, object extraction, contrast enhancement, and quality control checks, ensuring accurate identification and analysis of colonies while minimizing false positives. Additionally, the base method which we developed, is employed for object tracking, which involves object detection, state propagation across frames, association with existing objects, and object lifespan management, enabling accurate tracking and analysis over time. The object model used for tracking targets across frames consists of two main components: the representation of the target and the motion model used for predicting its position in the next frame. To estimate the target’s movement, a linear constant velocity model is employed, assuming that the inter-frame displacements remain consistent over time. This model operates independently of other objects and camera motion, ensuring accurate and reliable tracking of individual targets throughout the video sequence. In the target tracking process, when a detection is successfully associated with a target, the target’s state is updated by incorporating the information from the detected bounding box. This update involves optimizing the velocity components using a Kalman filter^70^ framework to achieve optimal estimation. However, if no detection is associated with the target in a particular frame, the target’s state is simply predicted without any correction using the linear velocity model. This hybrid approach enables accurate and continuous tracking of targets by updating their states whenever new detections are available, while also providing predictions when no new detections are found. The proposed method for tracking individual structures involves segmenting the structures and then performing Kalman filtering in image space, along with the Hungarian algorithm^71^ algorithm using bounding box overlap as an association metric, 1 to track and estimate their sizes over time. This integrated approach allows for accurate and reliable tracking of structures throughout the temporal sequence of images, providing valuable insights into their behavior and characteristics. Estimated size of colonies was converted to pixel unit and performed fitting time-resolved data analysis using an open-source R package QurvE^72^.

### Statistics and reproducibility

Statistical analysis was performed using Prism 9.5 or 10.0 (GraphPad software, San Diego, US). For experiments with 3 or more groups, data were analyzed by Kruskal–Wallis test with Dunn´s multiple comparisons test. Pairwise analysis in experiments performed with two experimental groups were done using non-parametric, two-tailed Mann–Whitney test or parametric, two-tailed Student´s *t*-test, as appropriate. Single-cell sorting was performed with *N* = 96–960 individual cells per pre-sorting culture. All pre-sorting culture represent completely independent biological replicates started with different colonies. All experiments have been repeated with at least 2 biological replicates showing similar outcomes.

### Specific experimental procedures for proof-of-principle studies with fluorescent reporter strain

#### Sample preparation for batch dilution

Overnight grown *sar*A-GFP reporter strain cells were harvested from TSAcm plates and resuspended in TSBcm liquid media to achieve an absorbance (A600) of 0.5. This sample is referred to as the undiluted culture. Tenfold serial dilutions were made in TSBcm for up to a dilution of 1e-11. 200 µl from each dilution was dispensed in 96-well plates (64 wells/dilution) and growth were monitored using BioSpa-Synergy instruments. Bacterial concentration per milli-liter of the undiluted batch culture was calculated by drop-dilution method by spotting 10 µl of cell suspension from each dilution on TSAcm. Colonies were counted 20 h post incubation at 37 °C.

#### Sample preparation for FACS

A fresh single colony of the *sar*A-GFP reporter strain grown overnight (pre-sorting culture) was suspended in PBS (D8537, Sigma) and mixed well using a vortexer. Suspended bacteria were sonicated for 5 min at room temperature (RT) using Branson 3510 water sonicator to break bacterial aggregates. The cells were diluted to ~1e7 cell suspension in 1 ml PBS, were stained with 0.067 mM propidium iodide (PI, B34954, Invitrogen) for 5 min at RT, and were sonicated for 1 min prior to flow cytometric analysis. Unstained cells were prepared analogously and were used to set up flow cytometer gates.

#### Flow cytometry and FACS-sorting

The blue laser (488 nm) was used to collect Forward scatter (FSC) and Side scatter (SCC) through photodiode, and GFP signals through 530/10 bandpass filter; The yellow-green laser (561 nm) was used to collect PI signals through 582/15 filter. Threshold operator or triggering threshold was set to 200 on SSC. PMT voltages were adjusted for optimal separation of different populations as 650 (FSC), 420 (SSC), 650 (GFP), and 850 (PI). The general gating strategy used in this is presented in Supplementary Fig. 2. Unstained cells were used to set up the gates for both GFP- and PI- positive and negative populations, respectively. First, 0.58, 0.79, and 1.3 µm beads (NPPS-4K, Spherotech) were used to adjust log intensities of side scatter (SSC) vs forward scatter (FSC) scales such that beads can be clearly separated from the buffer noise. The integrated value of area (*A*) is usually sufficient to resolve larger cell types such as immune cells, however, we used integrated value of height (*H*) for SSC and FSC which fits all events well within the plot area while clearly separating the buffer noise. SSC and FSC voltages (PMT) were adjusted for unstained cells as beads and cells of same sizes have different refractive indices^73^. The relationship between size beads and bacterial cell population is shown in Supplementary Fig. 2A, B. Beads smaller than 1 µm in size showed a wide FSC profile despite being uniform in size (Supplementary Fig. 2A-v) making it difficult to distinguish beads measuring 0.59 and 0.79 µm. The FSC profile of the bacterial showed size distribution within this range (Supplementary Fig. 2A-iii, 2B-iii). In contrast, the SSC-A profile showed a clear distinguish beads of different sizes. Resolving submicron particles is challenging using a conventional flow cytometer^74^ and SSC has been used to clearly resolve submicron particles^75^. SSC-A distribution of bacterial cells showed a narrow distribution which was clearly distinct from buffer noise albeit did not provide information about clustered cells. To resolve cell clustering, we used SSC-H versus SSC-A dot plot display which distinguished singlets from clumps but unable to differentiate buffer noise (Supplementary Fig. 2A-v, 2B-v, 2C-v). Therefore, we used both SSC-A histogram plot and SSC-H vs SSC-A dot plot display to sort single-cell events. We gated the P1 population (SSC^low-P1^) on SSC-A histogram while avoiding the visible clusters on SSC-H vs SSC-A dot plot. The population that represents clsuters on SSC-H vs SSC-A dot plot was gated as P2 population on SSC-A histogram and was set to capture events with the highest SSC-A signals (SSC^high-P2^). Note that SSC^high-P2^ population has two distinct subpopulations on SSC-H vs SSC-A dot plot, P2a and P2b (Supplementary Fig. 2C-v). P2a population is continuation of P1 population and likely contain singlets which we excluded from single cell sorting to prevent potential cell-size bias as this population might contain larger cells in various stages of division process. P2b population on the other hand represent cells that has form clusters. Relationships between different peaks in different plots is shown in Supplementary Fig. 2C.

The SSC^low-P1^ population was discriminated for the presence or absence of GFP by gating GFP+ or RSG+ population and for PI signal to eliminate dead cells (PI positive cells) on a dot plot of log PI-A and GFP-A intensities. Dot plot was split into four quadrants, as, Q1 (PI+, GFP-/RSG-), Q2 (PI+, GFP+/RSG+), Q3 (PI-, GFP-/RSG-), and Q4 (PI-, GFP+/RSG+). GFP/RSG fluorescence of the 4th quadrant (Q4) population was displayed on a histogram of counts and log GFP/RSG-A intensity. On this histogram, low, intermediate, and high GFP intensity populations (GFP/RSG^low^, GFP/RSG^int^, and GFP/RSG^high^, respectively) were gated as P4, P5 and/or P6 (Supplementary Fig. 2D).

#### Imaging flow cytometery analysis

Single- or multiple cells were sorted onto agar plates for CFU analysis or into 96-well plates for growth analysis in liquid culture, following the general procedures described above. Fluorescently labelled bacteria from the P4, P5 and/or P6 gates were also subjected to imaging flow cytometry. At least 60,000 events per gate were sorted using FACSAria and were used as input for imaging flow cytometry (ImageStreamX MkII, Amnis). Samples were run at low speed and high sensitivity setting; images were collected using 60× magnification. We collected GFP- signals using 488 nm laser into channel 2 (Ch02) through 528/35 bandpass filter. Speed beads (400041, Merck) were used for instrument alignment and were run constantly during image acquisition for focusing and camera alignment; Speed beads were visualized in channel 6 (Ch06). Channels 1 and 9 (Ch01 and Ch09) were used for bright field (BF) images and were used for camera alignment. Images were acquired using ISX.ex software and data were analyzed using IDEAS.exe (V 6.2) software. Using ISX, Ch02 intensities (log scale) of all events were displayed in a histogram. Speed beads bleach weak single up to the intensity of 1e4 in Ch02, therefore, intensities of 1e4 and higher were gated as GFP-positive signals, and 500–1000 GFP positive images were collected. Images were then imported to IDEAS where they were displayed as a function of Aspect ratio (*y*-axis) and Area (*x*-axis) in a dot plot. Singlets were gated between 0–50 *X*-axis and 0.87–1.0 *Y*-axis co-ordinates, while doublets were gated between 0–50 *X*-axis and 0.5–0.87 *Y*-axis co-ordinates. Events outside of these coordinates were considered as unresolved and were not included in data analysis (Supplementary Fig. 4). In contrast to the analysis presented on the *sar*A-GFP reporter strain, imaging flow cytometry analysis with WT cells labelled with RSG was not conclusive, since the fluorescent signal was not strong enough to distinguish bacterial cells from auto fluorescence of beads present in the calibration fluid.

#### Quantitative polymerase chain reaction (qPCR) and analysis

Duplicate samples of 150,000 *sar*A-GFP reporter cells from GFP^low^ and GFP^high^ populations were harvested using FACS and subjected to total DNA isolation using the ZymoBIOMICS DNA miniprep kit (D4304; Zymo Research) following manufacturer’s instructions. The plasmid DNA isolation was verified by PCR amplification using DreamTaq polymerase (K1071, ThermoFisher) and primer pair specific to the *GFP* gene present with-in the plasmid pCM29 (qGFP_F = 5′-gcactactggaaaactacctgt-3′ and qGFP_R = 5′-ctgtacataaccttcaggcatggca-3′). The chromosomal DNA isolation was verified using DreamTaq polymerase PCR and two different sets of primer pairs targeting *gyr*A (qGyrA-L = 5′-CGGTGATGATCGTCGTACAG-3′ and qGyrA-R = 5′-TACCTTGAACACCACGACCA-3′) and *Gro*EL (qGroEL-L = 5′-TGGCTAACACGTGCATCAAT-3′ and qGroEL-R = 5′-AAAAGCACCTGGTTTTGGTG-3′). The Primer pair targeting *gyr*A resulted in one specific band and one non-specific but faint band. The faint secondary band likely resulted from primers amplifying *gyr*B (Supplementary Fig. 3B). Consequently, for qPCR, we opted to use primers pairs targeting *gro*EL. qPCR was performed using LightCycler 174;96 (Roche) with Low ROX SYBER MasterMix (UL-LSMT-B0701, Takyon). Each sample was evaluated for *GFP* and *gro*EL in technical replicates (TR) and Cq values of TR were averaged. The plasmid copy number was defined as the ratio of plasmid to chromosomal DNA copies and was calculated using the formula 2^-ΔCq^, where –ΔCq represents the difference in quantification cycles between the plasmid gene (*GFP*) and the chromosomal gene (*gro*EL)^76^. qPCR data is presented in Fig. 2E and Supplementary Data 1 (sheet 2E).

### Specific experimental procedures for analysis of growth heterogeneity in strain *S. aureus* USA300 LAC (WT) colonies

#### Sample preparation

By default, single colonies of WT strain grown overnight (3 biological replicates) were suspended in PBS (D8537, Sigma) and mixed well using vortex mixer. Suspended bacteria were sonicated for 5 min at room temperature (RT) using Branson 3510 water sonicator to break bacterial clumps. Approximately 1e8 cells were stained with 0.1 µM *Bac*Light RedoxSensor Green (RSG, B34954, Invitrogen) for 10 min at 37 °C in dark. For RSG titration, 1e8 cells were stained with 0.00, 0.05, 0.1, 0.2 and 0.5 µM of RSG, as above (Supplementary Fig. 5). The cells were diluted to 1e7 cell suspension in 1 ml PBS, stained with 0.067 mM propidium iodide (PI, B34954, Invitrogen) for 5 min at RT, and sonicated for 1 min prior to flow cytometric analysis. Unstained cells were used to set up flow cytometer gates and were processed as above.

In experiments aimed at distinguishing populations originating from different locations within colonies aged 24 or 48 h, material was carefully harvested either from the center vs. edges of the colonies, or from the top vs. the bottom. These cells were prepared for flow cytometry analysis without RSG-labeling. PI^–^ single events were sorted from SSC^low-P1^-Q3 gate.

#### FACS-sorting and downstream analysis

The blue laser (488 nm) was used to collect Forward scatter (FSC) and Side scatter (SCC) through photodiode, and the RSG signal (FITC/GFP) through a 530/10 bandpass filter; The yellow-green laser (561 nm) was used to collect PI signals through a 582/15 filter. Threshold operator or triggering threshold was set to 200 on SSC. PMT voltages were adjusted for optimal separation of different populations as 650 (FSC), 420 (SSC), 650 (GFP), and 850 (PI). Single events were sorted into 96-well plates for growth analysis in TSB liquid culture following the general description above. Since RSG staining gave a broad distribution two subpopulations were gated, P4 with low RSG signal but high counts (RSG^low^) and P5 with high RSG signal but low counts (RSG^high^). RSG(–) subpopulation was also sorted (Supplementary Fig. 2D). We validated if PI and RSG staining on cell physiology post-sorting by comparing cell fitness of single sorted events from unstained and stained (PI, RSG, both PI & RSG) pre-sorting populations (Supplementary Fig. 3F); to do this, cells were sorted indiscriminately from the P1-subpopulation (Supplementary Fig. 2C-iv) based on their scattering profile.

### Specific experimental procedures for analysis of dormant growth variants elicited by low pH

#### Sample preparation

*S. aureus* individual colonies (3 biological replicates) were inoculated into the high glucose (4.5 g/L) DMEM media (with phenol red, L-glutamine, and sodium bicarbonate, without sodium pyruvate from Life Technologies) supplemented with 10% FBS. pH of this media was adjusted to pH 5.5 with citric acid. After incubation at 37 °C in 5% CO_2_ for 48 h samples were vortexed followed by centrifugation at 5000 rpm for 5 min at room temp. Supernatants were discarded and bacterial pellets were washed with pre-warmed PBS and the cell concentration was adjusted to McFarland 0.5. Next, samples were labelled with BacLight™ RedoxSensor™ Green (Invitrogen™) at a final concentration of 200 nM and incubated in dark at 37 °C for 10 min with moderate shaking. After 10 min, propidium iodide (PI) at final concentration 20 µM was added to the sample and incubated in the dark at 37 °C for 10 min.

#### FACS-sorting and downstream analysis

Samples were then analyzed by BD FACSAria III instrument (BD Biosciences). Neutral density (ND) filter 1.0 was used and the channel voltages were set as follows: Forward scatter (FSC, 200 V), side scatter (SSC, 350 V), threshold (FSC/SSC, 200) and a flowrate 1.0 to analyze the bacterial cell size, aggregation complexity, and background noise. Cellular fluorescence for RedoxSensor™ Green reagent was collected with blue laser 488 nm and 530/30 bandpass filter, whereas fluorescence from PI–stained samples was collected with a 561 nm excitation yellow-green laser and the (PE)-Texas Red (610/20 nm band-pass filter). A total of 50,000 events were recorded for post-data analysis. BD FACSDiva software V 9.0 was used to gate the target population of interest and sorting. Prior to target population gating, background noise was excluded from data by gating only cell population using FSC and SSC. Then RSG- stained live population cells were distinguished from PI stained dead population by their distinct FITC values (*x*-axis) against PE-TexRed values (*y*-axis) at logarithmic scale. After assessment of viability, the live RSG^+^, PI^–^ populations were gated and prepared for index sorting with single cell precision mode with a 70 μm nozzle. Sorting was performed on single well agar plate and 96-well plate with pre-added TSA for growth dynamic analyses in both liquid and solid media following the general procedures described above. Any post-analysis and visual representation of flow cytometry data was performed with Flowjo software. Data from agar were counted for events in three categories: (a) Regular colony, (b) Delayed growth (Colonies <1 mm at 24 h, colonies appearing at 48 h and 72 h), and (c) No colony. Colony size was measured using Fiji, an image processing package of ImageJ2 tool^77^. For liquid growth analysis, the cell fates were (a) Regular growth, (b) Delayed growth, and (c) No growth.

### Specific experimental procedures for antibiotic dependent colony variant vancomycin-intermediate *S. aureus* strain (VISA)

#### Sample preparation

Single colonies (3 biological replicates) of the vancomycin susceptible *S. aureus* strain ATCC 29213 and representative clinical MRSA-VISA strain Mu50 (ATCC 700699) were grown overnight in TSB at 37 °C. Prior to labeling with Vancomycin-BODIPY FL (Vanco-BFL), both *S. aureus* strains were grown to exponential phase and cell concentration was adjusted to McFarland 0.5. Next, samples were labelled with Vanco-BFL at a final concentration of 1 μg/ml and incubated in the dark at 37 °C for 30 min, before samples were additionally labelled with propidium iodide (PI) at final concentration 40 µM (adjusted for exponential phase) and incubated in the dark at 37 °C for 10 min prior to flow cytometry analysis.

#### FACS-sorting and downstream analysis

Vanco-BFL signal was collected with blue laser 488 nm and 530/30 bandpass filter, whereas fluorescence from PI–stained samples was collected with a 561 nm excitation yellow-green laser and the (PE)-Texas Red (610/20 nm band-pass filter). FSC and SSC was used to target the main cell events excluding the background noise. PI-negative cells were gated and sorted with index sorting (single cell precision mode activated from sorting layout in the BD FACSDiva software) onto Mueller Hinton agar (MHA) supplemented with different concentrations of vancomycin (0, 0.5, 2 or 8 μg/ml). Colony growth was evaluated after 24 and 48 h.

Cellular phenotypic profiling and backtracing analysis was performed with VISA cells sorted out onto MHA with 2 μg/ml vancomycin. After 48 h incubation at 37 °C, the cell fates were assessed as (a) regular growth, (b) colony size variant, and (c) no growth phenotypes. Indexed phenotypes were further traced back in flow cytometry data for finding ancestral relation. For all sorted events in the different cell fate groups, the cellular Vanco-BFL fluorescence intensity values were retrieved for group analysis in Prism 9.5.

For assessment of growth kinetics of VISA strain Mu50 in the presence or absence of vancomycin, cells were sorted onto MHA or MHA + 2 μg/ml vancomycin and colony growth were monitored using time-lapse imaging data as described above.

### Reporting summary

Further information on research design is available in the Nature Portfolio Reporting Summary linked to this article.

## Supplementary information

Supplementary Figs.

Description of Additional Supplementary Materials

Supplementary Movie 1

Supplementary Movie 2

Supplementary Dataset 1

Supplementary Dataset 2

Supplementary Dataset 3

Supplementary Dataset 4

Supplementary Dataset 5

Reporting Summary

## Data Availability

Source data is available in Supplementary Data 1–3.

## Code availability

All analysis performed with in house developed scripts for liquid media culture and time-lapse solid agar media kinetics are available at github.com. Code for ´single cell-derived growth analysis´ and ´time-lapse imaging of colony growth ´ were implemented in MATLAB (R2021a), The MathWorks, Natick, MA, USA have been deposited on Zenodo^78,79^: All scripts are free software and are free to redistribute and/or modify under the terms of the GNU General Public License as published by the Free Software Foundation, either version 3 of the License, or any later version.
