# Supplementary material for: Single-cell phenotypic profiling and backtracing exposes and predicts clinically relevant subpopulations in isogenic Staphylococcus aureus communities
Source: Commun Biol. 2024 Oct 1;7:1228. doi: 10.1038/s42003-024-06894-z (PMC11445386; doi:10.1038/s42003-024-06894-z)
Supplement: Supplementary file 1 — Supplementary Figs. [file 42003_2024_6894_MOESM1_ESM.pdf]

Single-cell phenotypic profiling and backtracing exposes and predicts clinically relevant subpopulations in isogenic *Staphylococcus aureus* communities.

Jonathan Hira[1,#], Bhupender Singh[1,#], Tirthankar Halder[1], Anel Mahmutovic[2], Clement Ajayi[1], Arif Ahmed Sekh[3], Kristin Hegstad[1,4], Mona Johannessen[1] and Christian S. Lentz[1,*]

[1] Centre for New Antibacterial Strategies (CANS) and Research Group for Host-Microbe Interactions, Department of Medical Biology, UiT – The Arctic University of Norway, 9037 Tromsø, Norway

[2]Early Biometrics & Statistical Innovation Data Science & AI AstraZeneca, Biopharmaceuticals RD AstraZeneca, Sweden

[3]XIM University, Bhubaneshwar, India

[4]Norwegian National Advisory Unit on Detection of Antimicrobial Resistance, Department of Microbiology and Infection Control, University Hospital of North Norway, 9038 Tromsø, Norway

[#] These authors contributed equally

* Corresponding author: Christian S. Lentz: Centre for New Antibacterial Strategies (CANS) and Research Group for Host-Microbe Interactions, Department of Medical Biology, UiT – The Arctic University of Norway, 9037 Tromsø, Norway. E-mail: christian.s.lentz@uit.no

Table of Contents

Supplementary Figure 1. 3
Supplementary Figure 2. 4
Supplementary Figure 3. 6
Supplementary Figure 4. 8
Supplementary Figure 5. 9
Supplementary Figure 6. 10
Supplementary Figure 7. 12
Supplementary Figure 8. 14
Supplementary References. 15

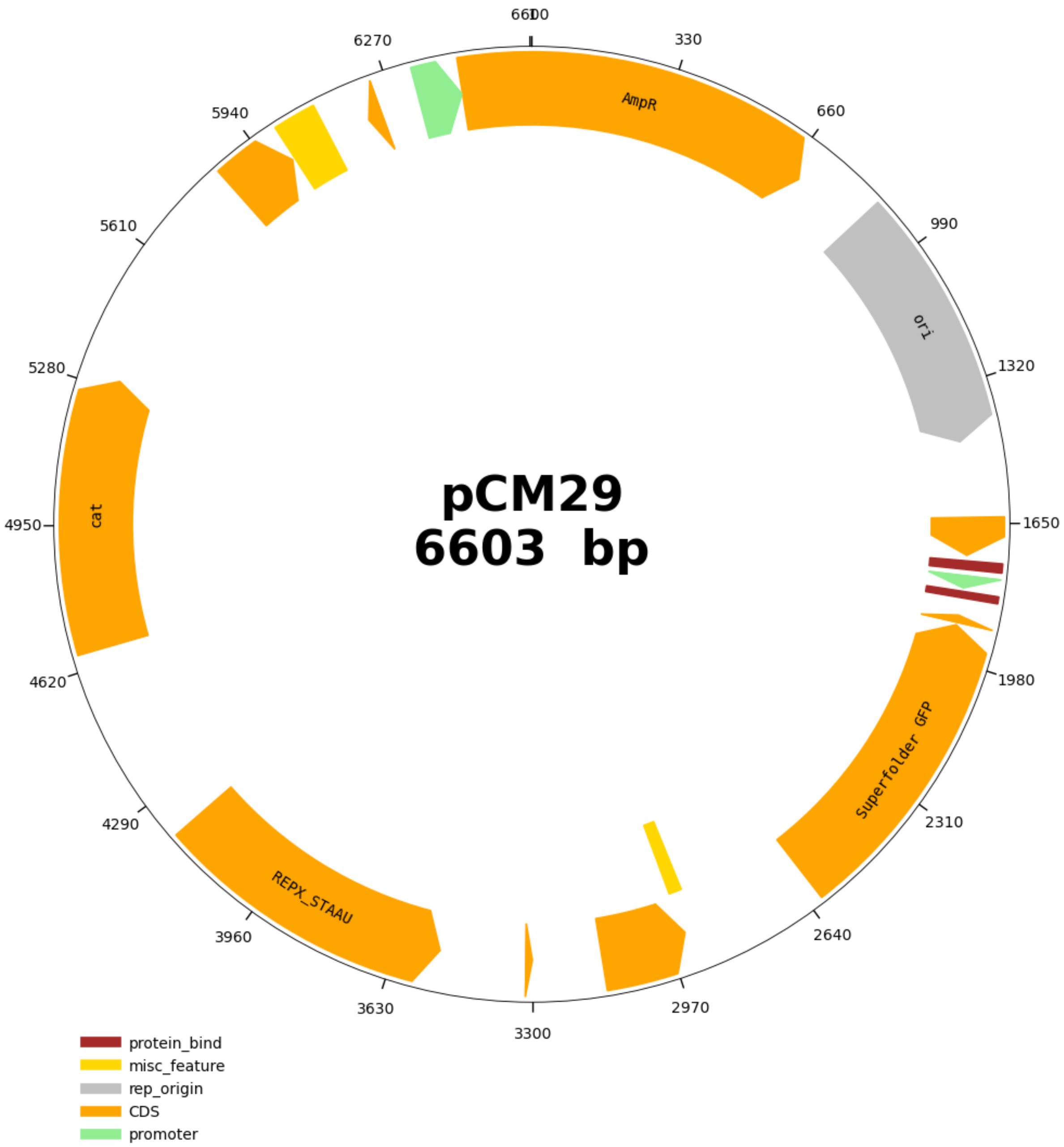

Supplementary Figure 1. Plasmid map of pCM29- P1*sar*A-GFP. The pCM29-P1-*sar*A-GFP plasmid was thankfully provided by Alexander Horswill. The vector pCM29 is providing chloramphenicol (cm) resistance and was constructed to express superfolder green fluorescent protein (sGFP) under *sar*A promoter P1 from *S. aureus* (P1*sar*A-GFP) [1]. It is a shuttle vector between *Escherichia coli* (*E. coli*) and *S. aureus*, and has pUC18 and pC194 backbone and origins of replication (*ori*). The plasmid was sequenced using nanopore techniques developed by Eurofins (https://eurofinsgenomics.eu/en/custom-dna-sequencing/eurofins-services/whole-plasmid-sequencing/). The plasmid sequence is provided in **Supplementary Data 5**. Although, the true copy number of plasmid in *S. aureus* strain is unknown, it is considered a high copy number plasmid [2]. pCM29 plasmid copy number evaluated in this study are presented in **Figure 2E** and data in **Supplementary Data 1 (sheet 2E)**.

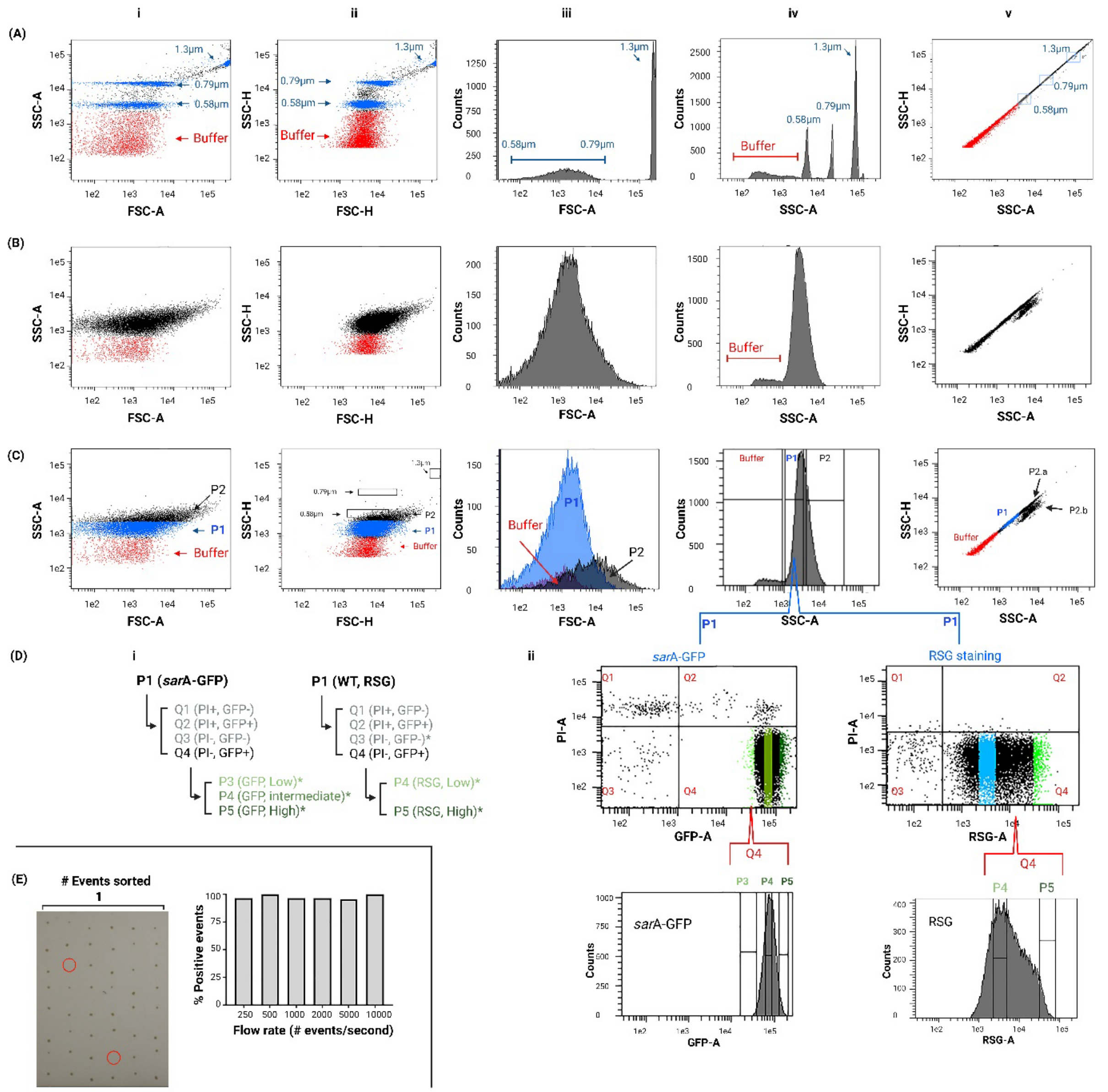

Supplementary Figure 2. Gating scheme and gate-hierarchy for experiments with *sar*A-GFP reporter and WT strain: (A) Flowcytometric profile of size beads. Bead events are indicated in blue and buffer noise is shown in red, (i) Size beads displayed as SSC-A versus FSC-A dot plot, (ii) displayed as SSC-H vs FSC-H dot plot, (iii) FSC-A histogram. Note that 0.58 and 0.79 µm sized beads are un-distinguishable, (iv) and SSC-A histogram. Note that all 0.58, 0.79 and 1.3 µm sized beads are distinguishable as well as separated from buffer noise, (v) SSC-H vs SSC-A correlation dot plot. (B) Flowcytometric profile of *sar*A-GFP reporter cells juxtaposed with the size bead profile displayed in row-A for comparison. Cells are shown in black and buffer noise in red, (i) cells displayed as SSC-A vs FSC-A, (ii) displayed as SSC-H vs FSC-H, (iii) FSC-A profile. Note the wide FSC-A distribution and lack of separation of buffer noise, (iv) and SSC-A profile. Note the narrow SSC-A profile separated from buffer noise, (v) SSC-H vs SSC-A correlation plot. Note cell cluster at high SSC range but lack of distinction from buffer noise. (C) Gating strategy for single-events sorting and different cell subpopulations, presented alongside row-B for comparison. Cells are shown in blue (subpopulation P1, SSC^{low}) and black (subpopulation P2, SSC^{high}), and buffer noise in red, (i) SSC-A vs FSC-A dot plot, (ii) SSC-H vs FSC-H dot plot, size beads location is marked for comparison, (iii) FSC-A histogram. Note that the buffer noise is hidden underneath cell population, and P1 and P2 subpopulation overlap, (iv) SSC-A histogram and bacterial populations

gating as P1 (SSC^{low}) and P2 (SSC^{high}), (v) SSC-H vs SSC-A dot plot. Note that the P2 subpopulation forms cell clusters and P1 subpopulation is placed well between buffer and clustered cells. (D) Downstream gating of P1 (SSC^{low-P1}) subpopulation for sorting. (i) overall gating hierarchy for *sar*A-reporter and WT cells stained with RSG. Sorted subpopulations are marked with *, (ii) P1 subpopulation displayed as PI-A and GFP-A to split P1 subpopulation as PI+GFP/RSG- (Q1), PI+GFP/RSG+ (Q2), PI-GFP/RSG- (Q3) and PI-GFP/RSG+ (Q4). GFP/RSG fluorescence of Q4 population was further displayed as GFP-A/RSG-A histogram and cell population was gated for their fluorescence intensity as low (*sar*A-GFP/RSG), intermediate (*sar*A-GFP) and high (*sar*A-GFP /RSG). (F) Evaluation of sorter efficiency at different flow rates. Photograph of CFUs emerging on agar plates following single sorted events. Red circles mark sorted events not leading to colony growth. Accompanied graph shows percent of successful single CFUs emerged following single event sorting at different flow rates. Data is presented in **Supplementary Data 3**. The figure was created with BioRender.

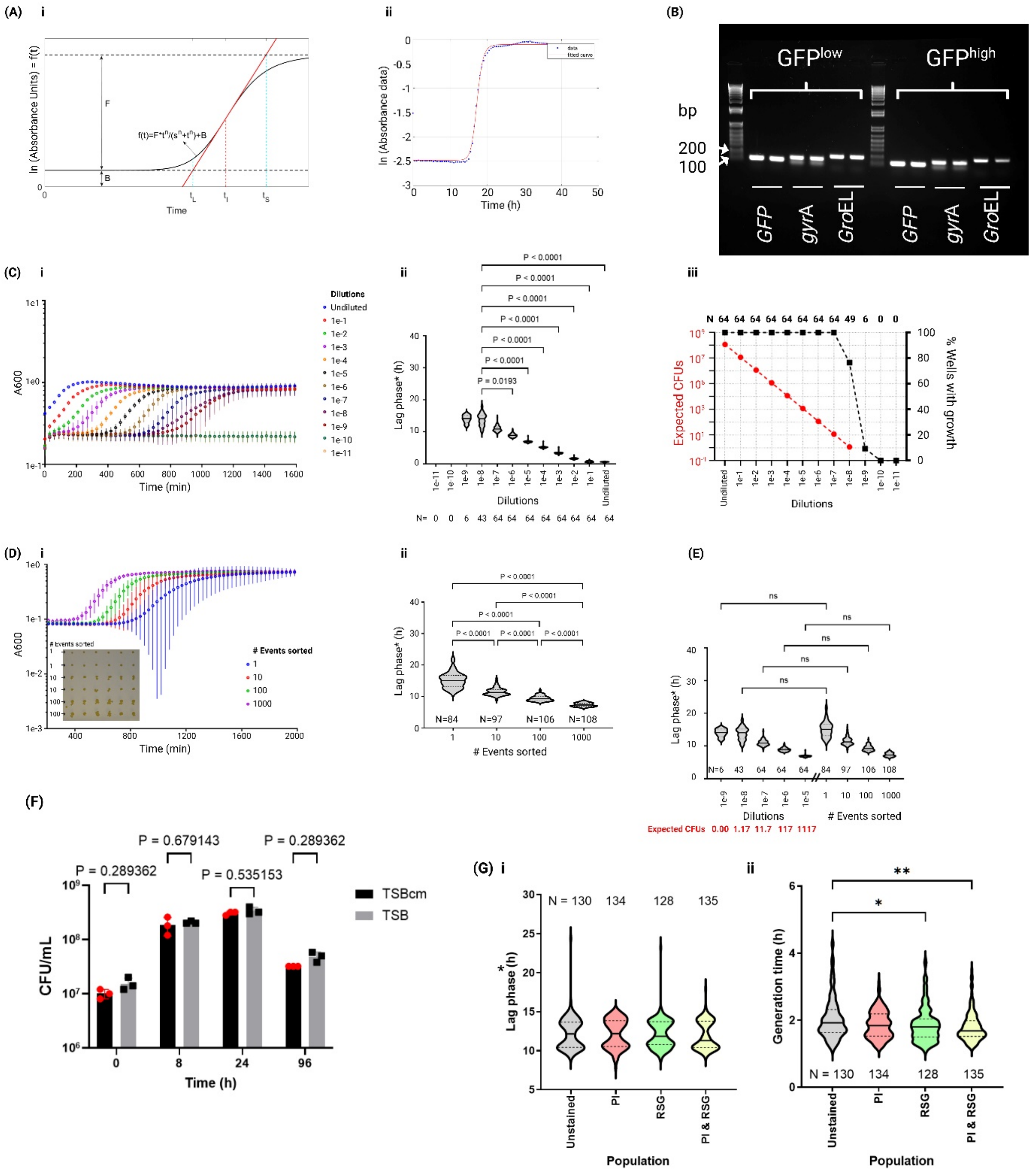

Supplementary Figure 3. Evaluation of single cell-derived liquid growth cultures. (A) Absorbance non-linear regression analysis. (i) Based on absorbance data expressed in log absorbance units (y-axis) versus time (x-axis), the duration of the lag phase (t_L), onset of the stationary phase (t_S) and the growth rate are defined in terms of a Hill function $f(t) = \frac{F*t^n}{(s^n+t^n)} + B$ where t is time, F the maximum absorbance relative to baseline B and s is the timepoint at which the absorbance level is at F/2 + B. The growth rate is defined as the slope of the tangent line of the Hill function at the inflection point, t_I, whereby t_L and t_S are defined as the intersection of the tangent line with the baseline B and the maximum absorbance level (F+B), respectively, (ii) Illustration of the outcome of the non-linear regression (red line) to actual data (blue dots). (B) Gel electrophoresis of PCR performed on DNA isolated in duplicate from cells sorted from GFP^{low} and GFP^{high} subpopulations targeting *GFP*, *gyr*A and *Gro*EL genes. A DNA ladder is placed in lanes preceding and following GFP^{low} samples. Note that the

*gyr*A PCR product show a faint secondary band beneath the main band. (C) Broth culture analysis of a batch-culture serially diluted by factors of 10 reaching a final dilution of 1e-11. (i) Growth curves represented by average absorbance measurements at 600 nm wavelength (A600) over a period of up to 48 h, with data presented for up to 1600 minutes, (ii) Violin plot showing the duration of the lag phase* (h) observed from cultures grown from different dilutions in TSBcm liquid media. Significant statistical comparison is provided only for the 1e-8 dilution against all other dilutions with N representing 0 to 64 post-dilution cultures. Raw data, calculations and statistical analysis of all other dilutions are presented in **Supplementary Data 3**. (iii) Expected CFUs in each well (red, left y-axis) and percent of wells showing growth (black, right y-axis) post dilution growth in different dilutions (x-axis). Each dilution was resuspended in 64 individual wells in 96-well plates. Data are presented in **Supplementary Data 3**. (D) Broth culture analysis of events sorted in multiples of 10 using FACS. (i) Growth curves represented by average A600 over a period of up to 48 h, with data presented between 200 to 2000 minutes. Inset: photograph of CFUs obtained on TSAcm agar media from 1-100 sorted events following overnight growth. (ii) Violin plot representing the duration of the lag phase* (h) from 4 biological pre-sort culture replicates of indicated sorted events in TSBcm liquid media with N representing 84 to 108 post-sorting cultures. Data are presented in **Supplementary Data 3**. (E) Violin plot, based on data from C-ii and D-ii showing a side by side comparison of the lag phase* (h) of cultures that derived from approximately similar numbers of cells dilution and cell sorting. Note the differences were not statistically significant. Data are presented in **Supplementary Data 3**. (F) Plasmid pCM29 stability in *S. aureus* USA300 LAC strain. Three independent colonies of *S. aureus* USA300 LAC carrying plasmid pCM29 were harvested from TSA agar media containing 10 µg/mL chloramphenicol (TSAcm) and were resuspended in TSB liquid media with (TSBcm) and without (TSB) 10 µg/mL chloramphenicol, and incubated over a period of 96 h at 37°C and 600 rpm shaking. Plasmid stability was determined by comparing growth of bacteria derived from TSBcm and TSB cultures on TSAcm agar plates. Samples from TSBcm and TSB growing cultures were drawn at 0, 8, 24 and 96 hours to determine CFUs. CFU/mL are presented on Y-axis over a period of 96 h on x-axis. Data are presented in **Supplementary Data 3.** (G) Impact of PI, RSG, PI & RSG staining on cell fitness. Violin plots representing (i) the duration of the lag phase* (h), and (ii) generation time (h) from 2 biological pre-sort culture replicates of single sorted events in TSB liquid media with N representing 128 to 135 post-sorting cultures. Cells were stained with indicated stains and were sorted based on scattering rather than fluorescence. Duration of lag phase* did not differ among stained and unstained cells, while RSG and double-stained cells showed slightly shorter generation time than unstained, but no PI stained cells. Data are presented in **Supplementary Data 3**. Statistical analysis in C-ii, D-ii, E and G were performed by Kruskal-Wallis test in combination with Dunn´s multiple comparison test, and in F was performed using unpaired t-test with Welch correction. Cross lines in all violin plots represent median value. The figure was created with BioRender.

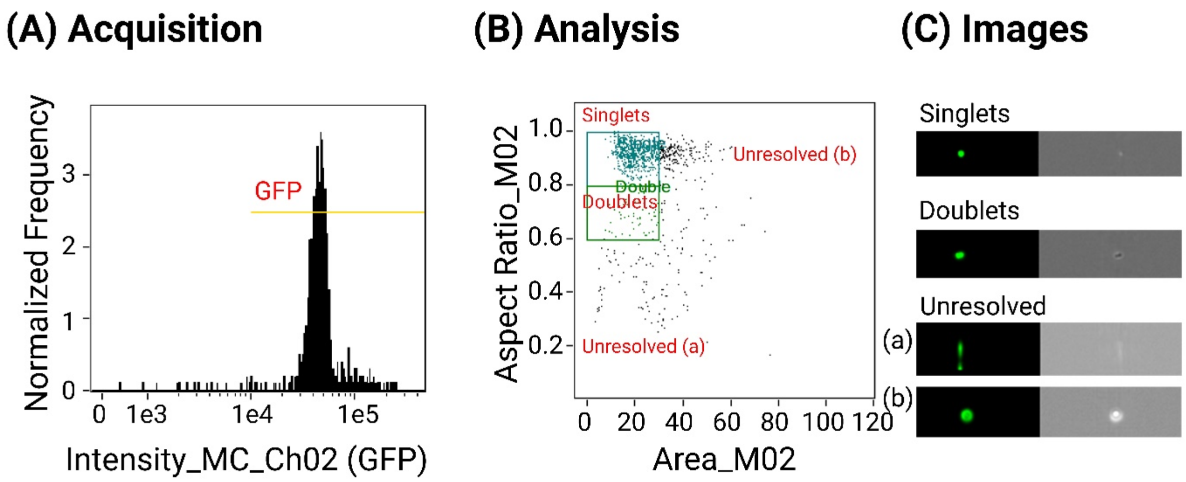

Supplementary Figure 4. ImageStream analysis of FACS-purified cells**.** (A) GFP intensity of all events displayed as normalized frequency and intensity of Ch02 (GFP channel). Events with GFP intensity higher than 1e4 were gated as GFP+ and data from these events were acquired. (B) GFP+ events displayed as aspect ratio and area. Events were gated as singlets (co-ordinates, X-axis 0-50, Y-axis 0.87-1.0) and doublets (co-ordinates, X-axis 0-50, Y-axis 0.5-0.87). The remainder of the events was considered as unresolved and were not included in analysis. Events were considered unresolved when they either failed to form a circular shape (a) or to focus properly (b). (C) GFP (left) and bright field (right) images of selected cells from singlets, doublets, and unresolved populations. The figure was created with BioRender.

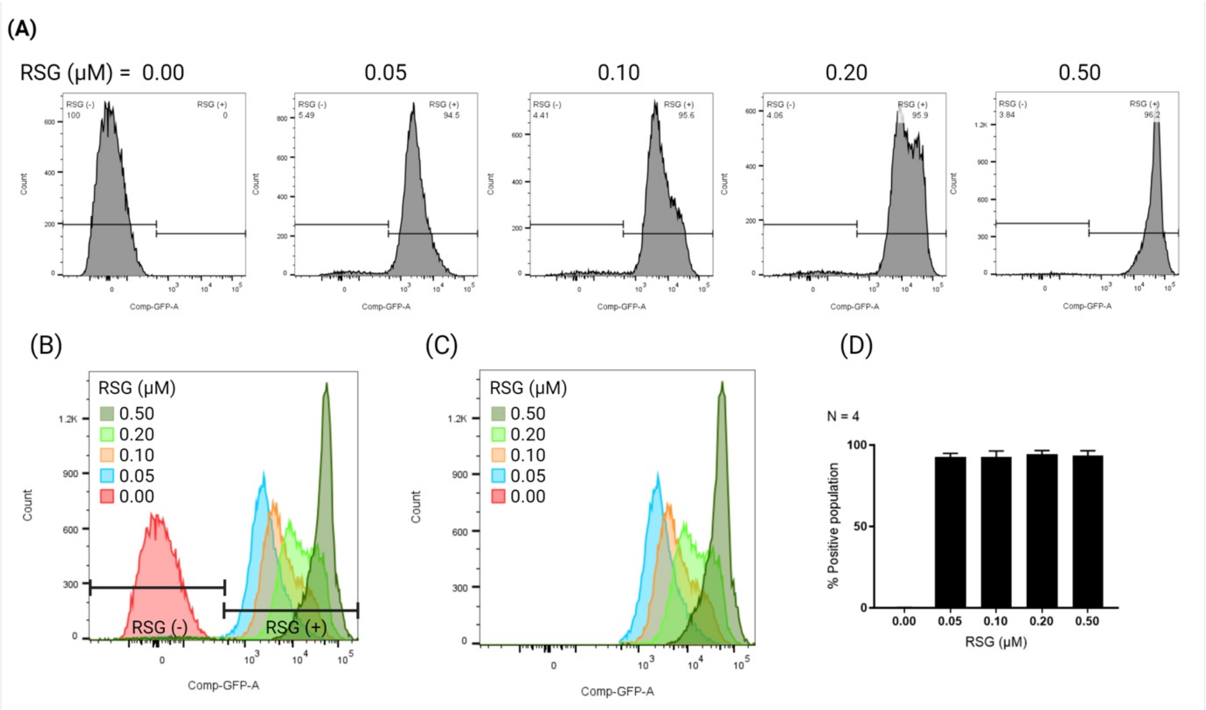

Supplementary Figure 5. RSG titration of *S. aureus* USA300 LAC (WT) cells**.** Flow cytometry histograms of a WT cell colony stained with different concentrations of RSG. (A) Individual histograms with RSG$^{(-)}$ and RSG$^{(+)}$ gating. (B) Overlay of histograms shown in A. (C) Overlay of only RSG$^{(+)}$ population stained with different RSG concentrations. (D) Combined analysis of the RSG-positive population (N=4 biological replicates). Data are presented in **Supplementary Data 3 (sheet S5D)**. The figure was created with BioRender.

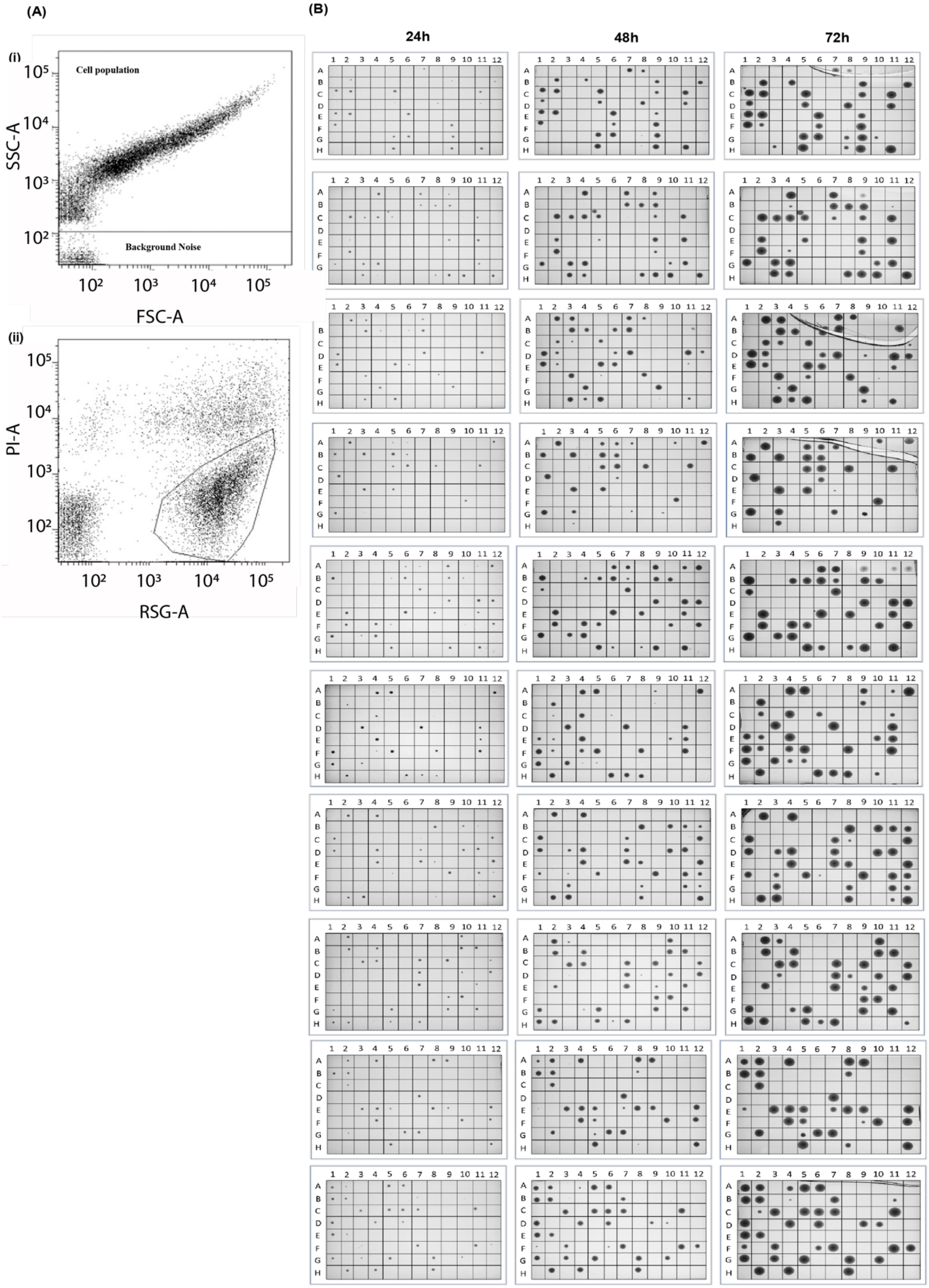

Supplementary Figure 6. Colony growth heterogeneity of *S. aureus* LAC cultures grown at low pH.

After cultivation in DMEM, pH5.5 for 48 h, *S. aureus* USA300 LAC cells were labeled with RSG and PI , followed by flow cytometry. 960 RSG(+), PI(-) cells were sorted on rectangular plates TSA plates (10 plates with 960 cells each. Colony growth was monitored at 24 h, 48 h, and 72 h. (**A**) Flow cytometry

dot plot represents (i) Gating of all cellular event excluding background noise in FSC-A vs. SSC-A plot. (ii) Gated population of RSG(+)-PI(-) positive live cell population used for single-cell sorting. **(B)** An overview of colony growth on agar plate at 24h, 48h and 72h. The figure shows images of 10 different plates (rows) per timepoint (column). The data was used for backtracing analysis shown in **Figure 4**.

Supplementary Figures

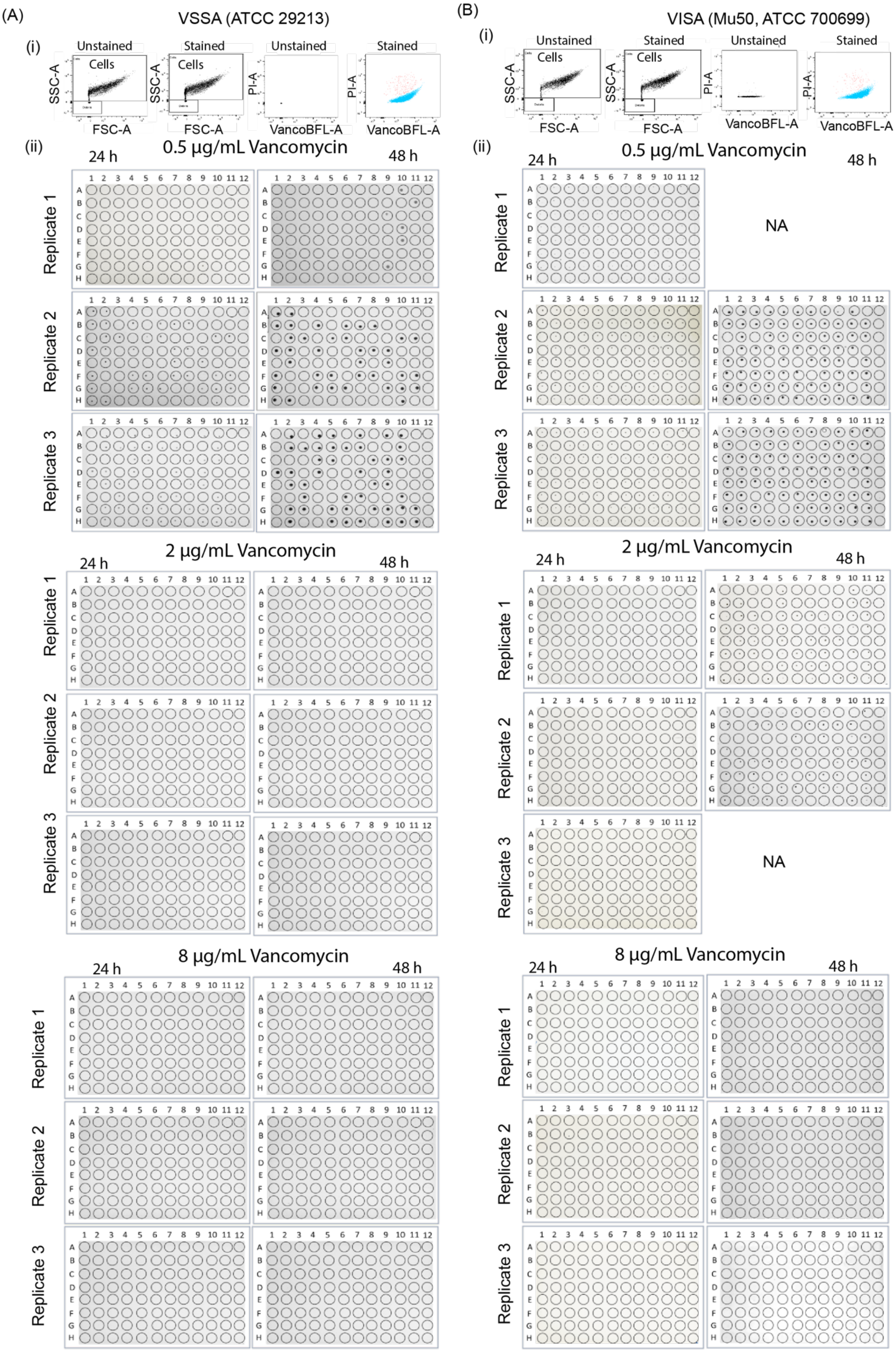

Supplementary Figure 7. Cellular phenotypic profiling coupled to single cell-derived vancomycin susceptibility testing. (A) VSSA strain ATCC 29213 and (B) VISA Mu50 ATCC 700699 cells were labelled with Vanco-BFL and propidium iodide for flow cytometry analysis and single-cell sorting for growth analysis in the presence of different concentrations of vancomycin (0, 0.5, 2 or 8 mg/mL). (i) Flow cytometry dot plot represents gating of all cellular events under stained or unstained condition, and Vanco-BFL positive (blue) and PI positive (putatively dead) population (red). (ii) Colony growth analysis after sorting of single cells onto MHA plates supplemented with the indicated different concentrations.

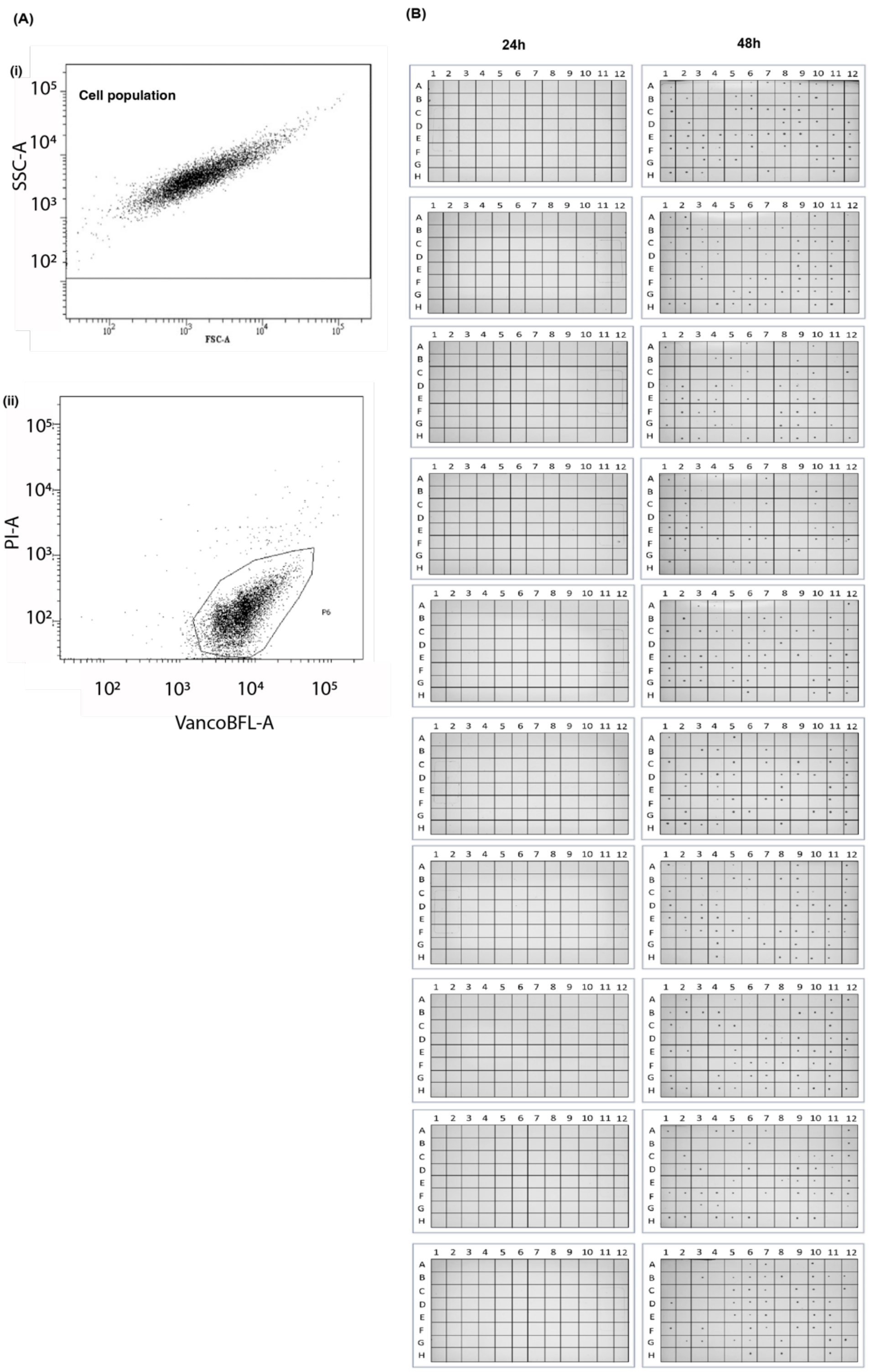

Supplementary Figure 8. Growth heterogeneity of *S. aureus* VISA strain Mu50 at 0.25x vancomycin. VISA Mu50 ATCC 700699 cells were labelled with VancoBFL and PI and 960 PI(-) single cells were FACS-sorted for growth analysis on agar at 0.25x MIC vancomycin. A) The flow cytometry dot plot illustrates (i) the gating of all cellular events, excluding background noise, and (ii) the gated population of VancoBFL-positive, PI-negative cells used for sorting of live cells. (B) Photographic images of 10 MHA plates (rows) supplemented with 0.25x vancomycin showing colony at 24 and 48 h post sorting (columns). The data was used for backtracing analysis shown in **Figure 5**.

Supplementary References

1. Pang, Y.Y. et al. agr-Dependent interactions of Staphylococcus aureus USA300 with human polymorphonuclear neutrophils. *J Innate Immun* **2**, 546-59 (2010).
2. de Jong, N.W., van der Horst, T., van Strijp, J.A. & Nijland, R. Fluorescent reporters for markerless genomic integration in Staphylococcus aureus. *Sci Rep* **7**, 43889 (2017).
