# Supplementary material for: Single-cell phenotypic profiling and backtracing exposes and predicts clinically relevant subpopulations in isogenic Staphylococcus aureus communities
Source: Commun Biol. 2024 Oct 1;7:1228. doi: 10.1038/s42003-024-06894-z (PMC11445386; doi:10.1038/s42003-024-06894-z)
Supplement: Supplementary file 2 — Description of Additional Supplementary Materials [file 42003_2024_6894_MOESM2_ESM.pdf]

Description of Additional Supplementary Files

File name: Supplementary Data 1

Description: The source data behind Figures 2 and 3 in the paper

File name: Supplementary Data 2

Description: The source data behind Figures 4 and 5 in the paper

File name: Supplementary Data 3

Description: The source data behind Supplementary Figures 2, 3 and 5.

File name: Supplementary Data 4

Description: Instructions for using 'Single cellderived growth analysis' script

File name: Supplementary Data 5

Description: Plasmid pCM29 sequence FASTA file

File name: Supplementary Movie 1

Description: Time-lapse colony growth analysis of sorted Mu50 cells on MH agar without vancomycin

File name: Supplementary Movie 2

Description: Time-lapse colony growth analysis of sorted Mu50 cells on MH agar supplemented with 0.25x MIC vancomycin
