# Supplementary material for: Single-cell phenotypic profiling and backtracing exposes and predicts clinically relevant subpopulations in isogenic Staphylococcus aureus communities
Source: Commun Biol. 2024 Oct 1;7:1228. doi: 10.1038/s42003-024-06894-z (PMC11445386; doi:10.1038/s42003-024-06894-z)
Supplement: Supplementary file 10 — Reporting Summary [file 42003_2024_6894_MOESM10_ESM.pdf]

nature portfolio

Corresponding author(s): Christian S. Lentz

Last updated by author(s): Sep 3, 2024

Reporting Summary

Nature Portfolio wishes to improve the reproducibility of the work that we publish. This form provides structure for consistency and transparency in reporting. For further information on Nature Portfolio policies, see our Editorial Policies and the Editorial Policy Checklist.

Statistics

For all statistical analyses, confirm that the following items are present in the figure legend, table legend, main text, or Methods section.

n/a	Confirmed	
☐	☒	The exact sample size (*n*) for each experimental group/condition, given as a discrete number and unit of measurement
☐	☒	A statement on whether measurements were taken from distinct samples or whether the same sample was measured repeatedly
☐	☒	The statistical test(s) used AND whether they are one- or two-sided<br>*Only common tests should be described solely by name; describe more complex techniques in the Methods section.*
☒	☐	A description of all covariates tested
☐	☒	A description of any assumptions or corrections, such as tests of normality and adjustment for multiple comparisons
☐	☒	A full description of the statistical parameters including central tendency (e.g. means) or other basic estimates (e.g. regression coefficient) AND variation (e.g. standard deviation) or associated estimates of uncertainty (e.g. confidence intervals)
☐	☒	For null hypothesis testing, the test statistic (e.g. *F*, *t*, *r*) with confidence intervals, effect sizes, degrees of freedom and *P* value noted<br>*Give P values as exact values whenever suitable.*
☒	☐	For Bayesian analysis, information on the choice of priors and Markov chain Monte Carlo settings
☒	☐	For hierarchical and complex designs, identification of the appropriate level for tests and full reporting of outcomes
☒	☐	Estimates of effect sizes (e.g. Cohen's *d*, Pearson's *r*), indicating how they were calculated

Our web collection on statistics for biologists contains articles on many of the points above.

Software and code

Policy information about availability of computer code

Data collection	Custom code (Single-cell-Time-Lapse-Imaging): https://github.com/HMIUiTL9/Colony-growth-time-lapse-Imaging.git
Data analysis	Quantitative growth curve Evaluation (QurvE) open-source, R package (https://nicwir.github.io/QurvE/)<br>Custom code (Single-cell-derived-growth-curve): https://github.com/HMIUiTL9/Single-cell-derived-growth-curve.git

For manuscripts utilizing custom algorithms or software that are central to the research but not yet described in published literature, software must be made available to editors and reviewers. We strongly encourage code deposition in a community repository (e.g. GitHub). See the Nature Portfolio guidelines for submitting code & software for further information.

Data

Policy information about availability of data

All manuscripts must include a data availability statement. This statement should provide the following information, where applicable:

- Accession codes, unique identifiers, or web links for publicly available datasets
- A description of any restrictions on data availability
- For clinical datasets or third party data, please ensure that the statement adheres to our policy

Source data and calculations are provided in Supplementary Data 1-6.

Research involving human participants, their data, or biological material

Policy information about studies with human participants or human data. See also policy information about sex, gender (identity/presentation), and sexual orientation and race, ethnicity and racism.

Reporting on sex and gender	n/a
Reporting on race, ethnicity, or other socially relevant groupings	n/a
Population characteristics	n/a
Recruitment	n/a
Ethics oversight	n/a

Note that full information on the approval of the study protocol must also be provided in the manuscript.

Field-specific reporting

Please select the one below that is the best fit for your research. If you are not sure, read the appropriate sections before making your selection.

☒ Life sciences ☐ Behavioural & social sciences ☐ Ecological, evolutionary & environmental sciences

For a reference copy of the document with all sections, see nature.com/documents/nr-reporting-summary-flat.pdf

Life sciences study design

All studies must disclose on these points even when the disclosure is negative.

Sample size	No sample size calculation was performed. Sample size was 3 or larger for all experiments except quantification of % grown cultures which was derived from n=2 independent experiments. For single-cell derived studies N >40.
Data exclusions	No data was excluded.
Replication	All results were replicated in at least 2 independent experiments and all attempts at replication of the results were successful.
Randomization	Not relevant, quantitative in vitro study only
Blinding	Not relevant, quantitative in vitro study only

Reporting for specific materials, systems and methods

We require information from authors about some types of materials, experimental systems and methods used in many studies. Here, indicate whether each material, system or method listed is relevant to your study. If you are not sure if a list item applies to your research, read the appropriate section before selecting a response.

Materials & experimental systems

n/a	Involved in the study	
☒	☐	Antibodies
☒	☐	Eukaryotic cell lines
☒	☐	Palaeontology and archaeology
☒	☐	Animals and other organisms
☒	☐	Clinical data
☒	☐	Dual use research of concern
☒	☐	Plants

Methods

n/a	Involved in the study	
☒	☐	ChIP-seq
☐	☒	Flow cytometry
☒	☐	MRI-based neuroimaging

Plants

Seed stocks	n/a
Novel plant genotypes	n/a
Authentication	n/a

Flow Cytometry

Plots

Confirm that:

☒ The axis labels state the marker and fluorochrome used (e.g. CD4-FITC).

☒ The axis scales are clearly visible. Include numbers along axes only for bottom left plot of group (a 'group' is an analysis of identical markers).

☒ All plots are contour plots with outliers or pseudocolor plots.

☒ A numerical value for number of cells or percentage (with statistics) is provided.

Methodology

Sample preparation	Bacterial cells, fluorescent or otherwise, were harvested in PBS buffer. Where ever applicable, cells were stained with either Fl. Dye (PI) or Fl. Probe (RSG, Van-Fl) prior to flowcytometer. Detailed sample preparation for different assays is presented in supplementary Online method.
Instrument	BD FACSAria III Cell Sorter (2015-07-14T, BD Biosciences) and ImageStream®X Mark II (Amnis)
Software	BD FACSDiva software V 9.0 and FlowJo software (V 10.8.1_CL), IDEAS.exe (V 6.2
Cell population abundance	Cells were sorted to obtained primarily single cell. Sorting efficiency was determined by observing 1. CFU formation per sorting event, 2. comparative analysis of growth kinetics of single and multiple sorting events and direct observation using Imagestream. Figures 2-3, Supplementary Figure 2, 3,4,6,7 and 8 represent the detailed outcome of post-sort fractions.
Gating strategy	For proof of principle studies:<br>1. For FACS aria: Unstained or non-fluorescent cells were used to set the threshold between positive and negative populations. SSC display was first used to separate cell population from buffer noise, followed by displaying cells and gating different populations in a dot plot based on their fluorescent profiles (PI, y-axis; GFP, x-axis). GFP positive and PI negative population was further gated based on fluorescence intensities. Detailed gating scheme is presented in Figure S1 and in supplementary online method.<br>2. For Imagestream: Cells were displayed on a histogram depicting fluorescence intensity; Cells with Fl. intensity (x-axis) higher than 1e4 were gated and further displayed on a dot plot as aspect ratio (y-axis) and area (x-axis) to differentiate singlets and doublets. A detailed gating scheme is presented in Figure S3 and in supplementary online method.

☒ Tick this box to confirm that a figure exemplifying the gating strategy is provided in the Supplementary Information.
